# Supplementary material for: Structural basis for distinct inflammasome complex assembly by human NLRP1 and CARD8
Source: Nat Commun. 2021 Jan 8;12:188. doi: 10.1038/s41467-020-20319-5 (PMC7794362; doi:10.1038/s41467-020-20319-5)
Supplement: Supplementary file 1 — Supplementary Information [file 41467_2020_20319_MOESM1_ESM.pdf]

Structural basis for distinct inflammasome complex assembly by human NLRP1 and CARD8

Supplementary Information

Supplementary Figures 1-11
Supplementary Table 1

Supplementary Figure 1-11

Supplementary Figure 1. Additional data for the oligomerization of NLRP1 constructs

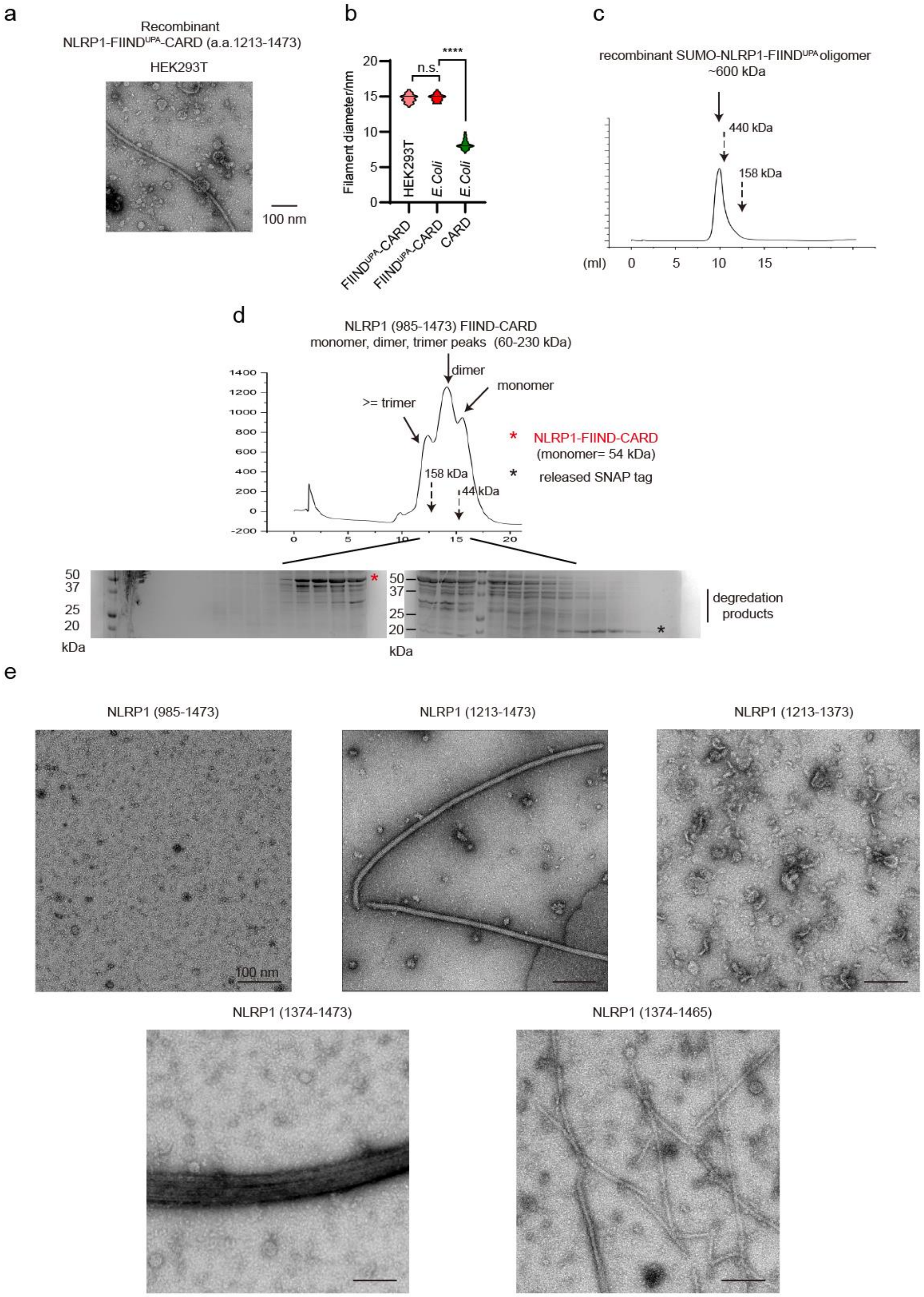

a. Negative EM images of NLRP1-FIND^{UPA}-CARD filaments purified from HEK293T cells.
b. Dimensions of NLRP1-FIIND^{UPA}-CARD and NLRP1-CARD filaments, based on negative stain EM images, ~100 individual measurements. P-value was calculated with One-way ANOVA.
c. Size exclusion profile of purified SUMO-NLRP1-FIIND^{UPA} on Superdex S200 increase column.
d. Size exclusion profile and SDS-PAGE analysis of NLRP1-FIIND^{fl}-CARD (a.a. 985-1473) construct. Unlike FIIND^{UPA}-CARD, FIIND^{fl}-CARD appears to be a monomer-dimer mixture, instead of a 15-20 mer mixture.
e. Negative stain EM images of NLRP1 constructs prepared from *E.Coli* sources.

Supplementary Figure 2. Additional data for the analyzing the importance of the linker length in NLRP1 and CARD8 signaling

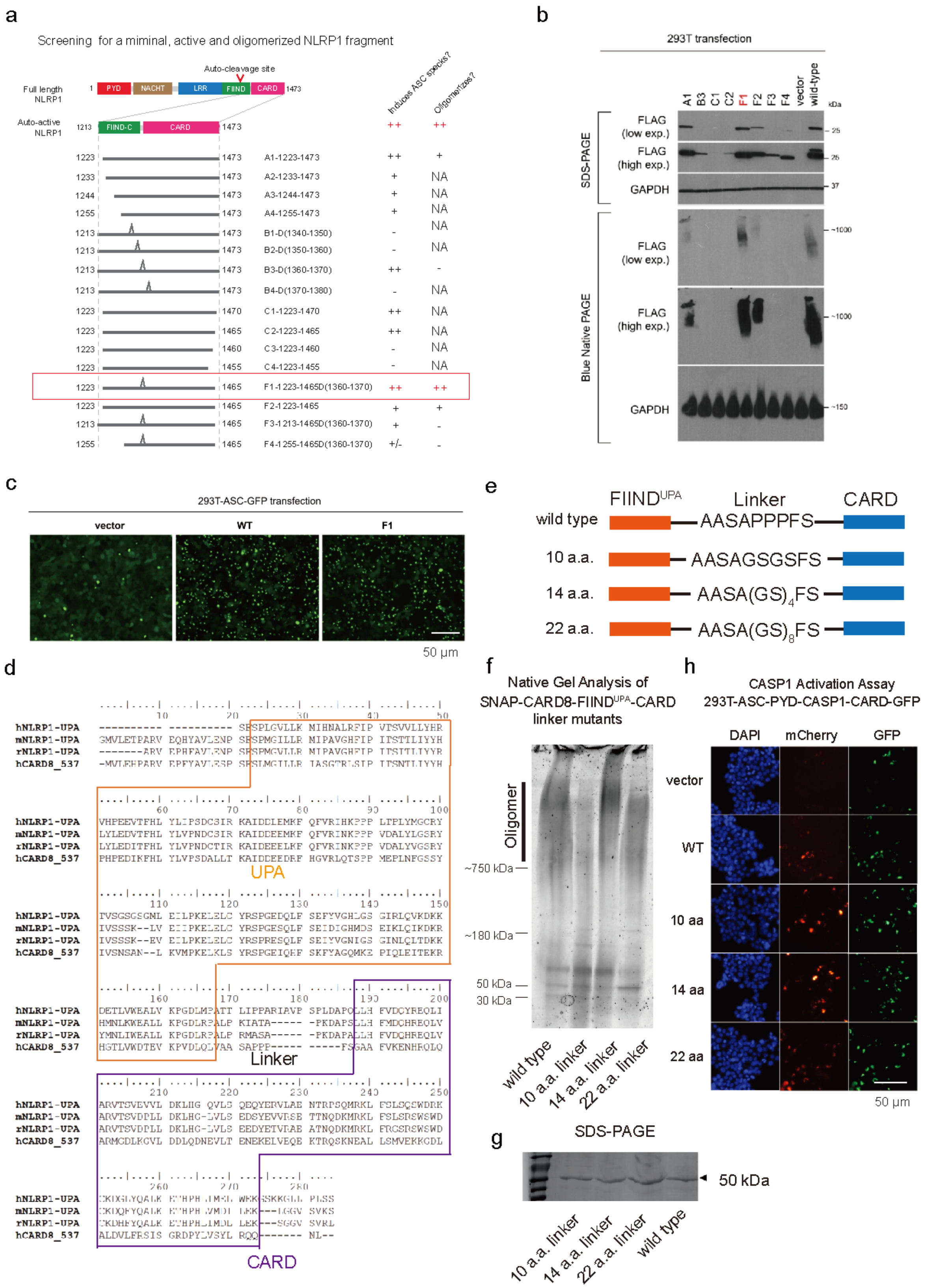

a. NLRP1 constructs boundary and linker truncation analysis, using ASC speck formation as read out.
b. Oligomerization, analyzed using native gel and probed by western blot, of selected NLRP1 constructs to validate the importance of flexible regions in NLRP1.
c. Raw images of ASC speck formation induced by selected NLRP1 mutants.
d. Sequence alignment of human, mouse and rat NLRP1, as well as human CARD8.
e. CARD8 linker mutants (SNAP-FIIND^{UPA}-CARD) design.
f. CARD8 linker mutants oligomerization analyzed by native gel.
g. CARD8 linker mutants oligomerization analyzed by SDS-PAGE.
h. CARD8 linker mutants analyzed by CASP1 speck formation.

Supplementary Figure 3. Additional data for the oligomerization of CARD8 constructs

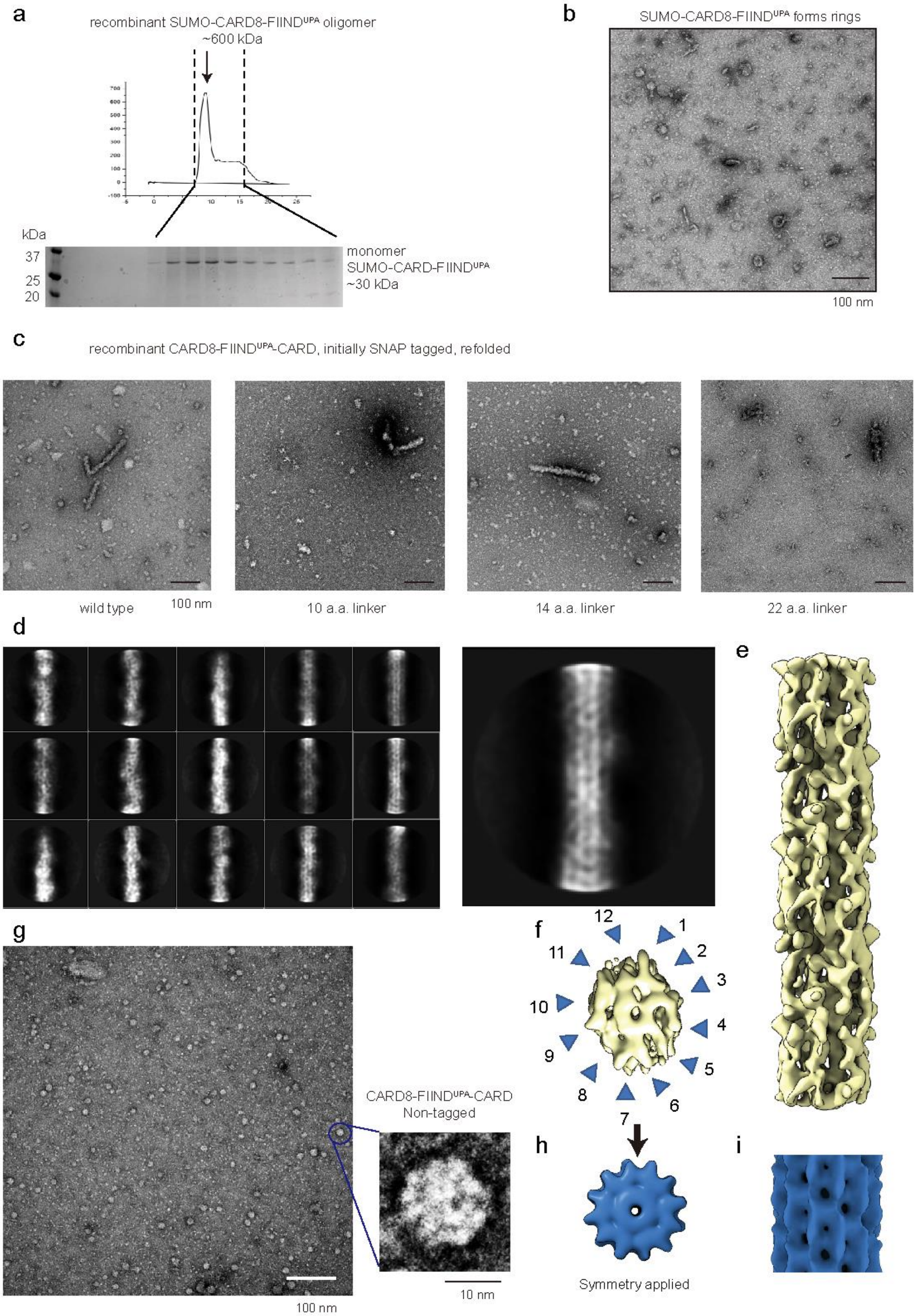

a. Size exclusion and SDS-PAGE profile of purified SUMO-CARD8-FIIND^{UPA}, Superdex S200 increase column.
b. Negative EM images of CARD8-FIND^{UPA} ring-complexes purified from *E.Coli.*
c. Negative EM images of CARD8-FIND^{UPA}-CARD complexes, wild type and the linker mutants.
d. Representative 2D class average images of CARD8-FIND^{UPA}-CARD.
e. 3D reconstruction of CARD8-FIND^{UPA}-CARD filament without imposing any symmetry.
f. Top view of reconstruction CARD8-FIND^{UPA}-CARD revealed a potential 11-12 unit per turn symmetry that is similar to NLRP1-FIND^{UPA}-CARD.
g. Negative stain EM images of CARD8-FIND^{UPA}-CARD when expressed without any fusion tags. A zoomed in view of a complex resembled the overall shape of the top view of helical reconstructed CARD8-FIND^{UPA}-CARD filament.
h. Top view of symmetry applied helical reconstruction of CARD8-FIND^{UPA}-CARD.
i. Side view of symmetry applied helical reconstruction of CARD8-FIND^{UPA}-CARD.

Supplementary Figure 4. Additional data for NLRP1-CARD filament analysis

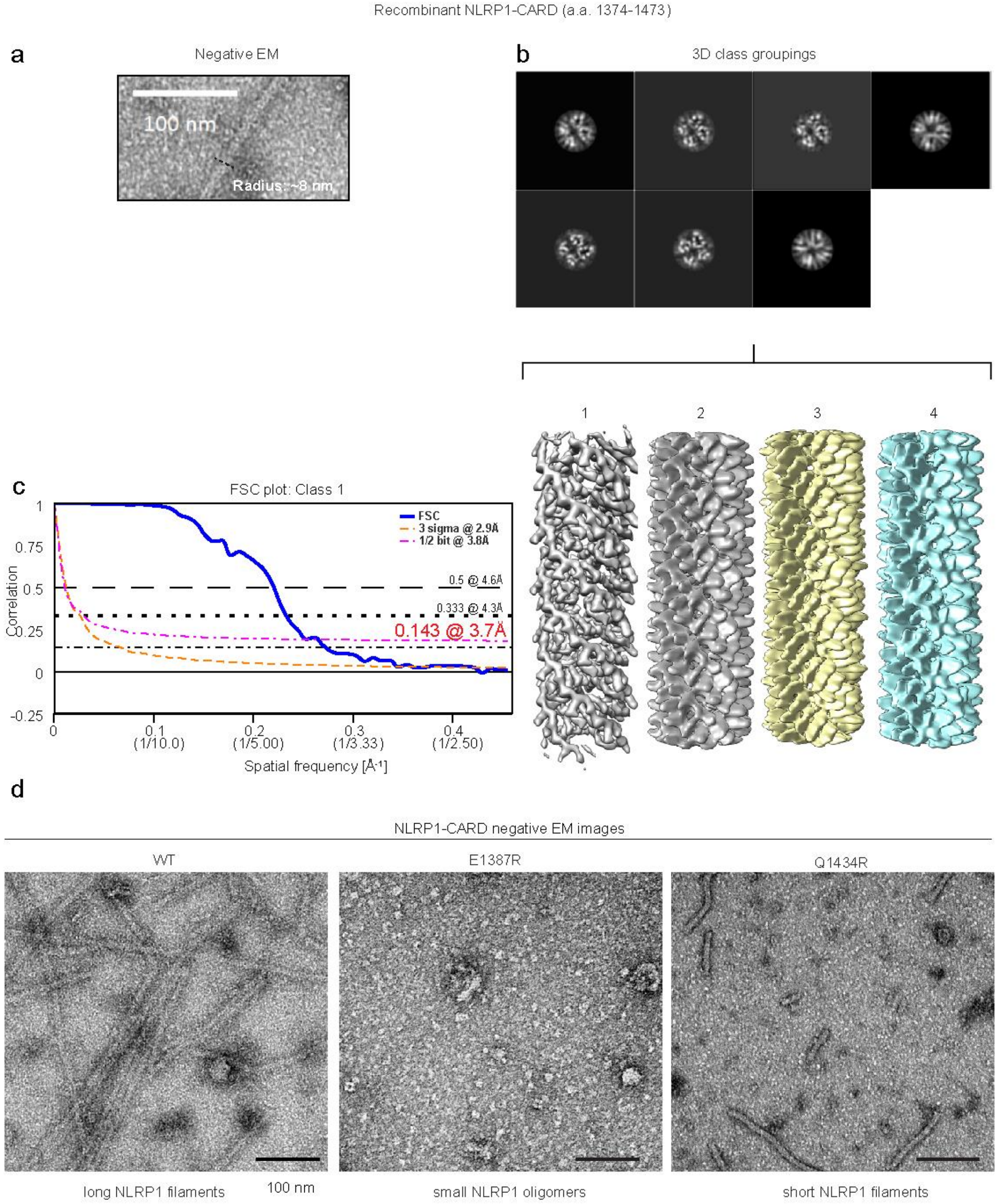

a. Representative dimensions of NLRP1-CARD filaments.
b. 3D densities of 4 groups of NLRP1-CARD filaments after helical symmetry was applied.
c. Gold standard FSC plot of the final post-refinement NLRP1-CARD filament density (EMD-9943).
d. Negative stain EM images of recombinant wild-type NLRP1-CARD, NLRP1-CARD^{E1387R} and NLRP1-CARD^{Q1434R}.

Supplementary Figure 5. Additional data for NLRP1-CARD and CARD8-CARD complex densities

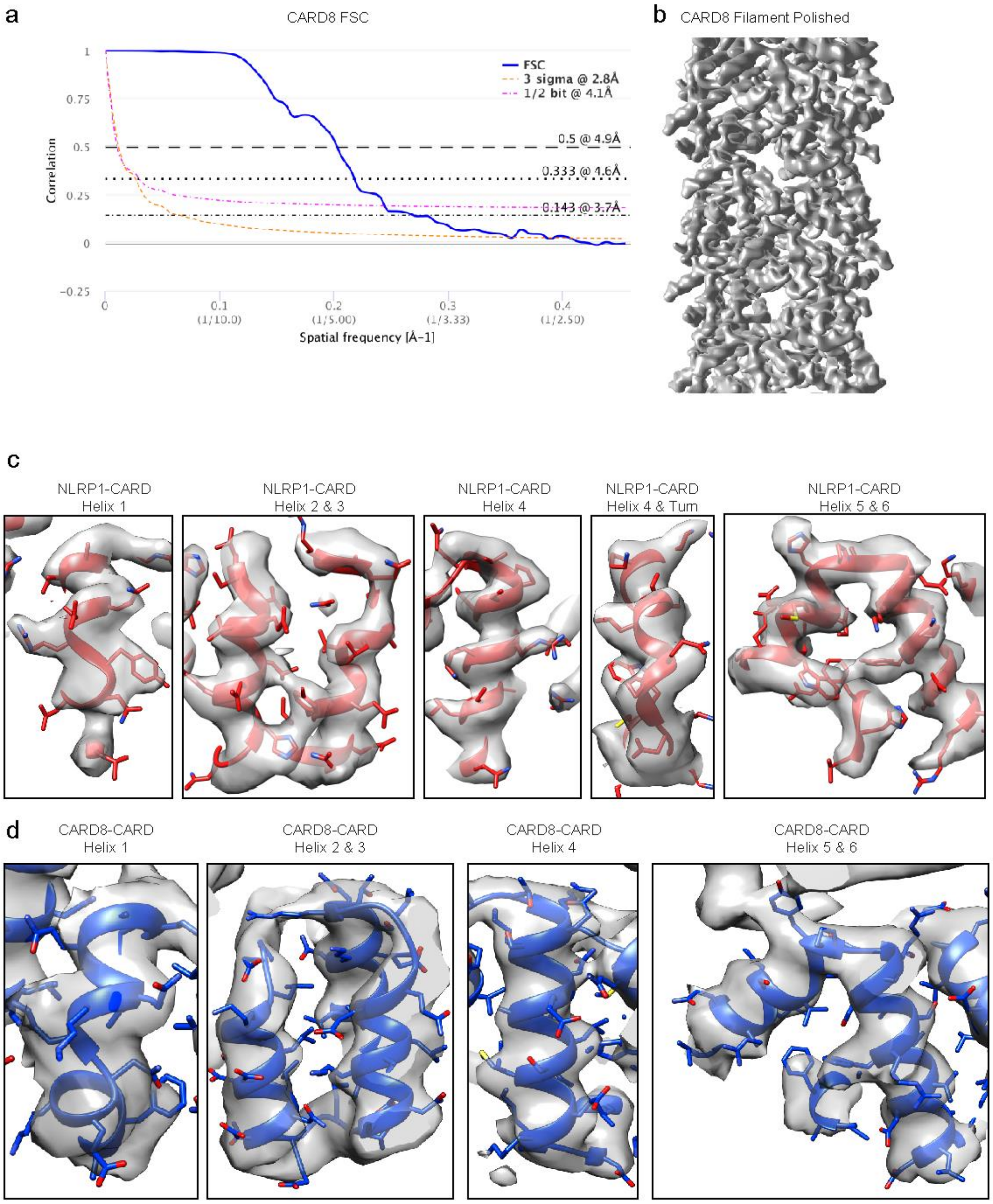

a. Gold standard FSC plot of final refined CARD8-CARD filament density (EMD-9948).
b. Post-refinement 3D density of CARD8-CARD filaments.
c. Assignment of individual residues and α-helices in NLRP1-CARD structure.
d. Assignment of individual residues and α-helices in CARD8-CARD structure.

Supplementary Figure 6. Additional data for ASC-CARD, CASP1-CARD and NLRC4-CARD structural analysis

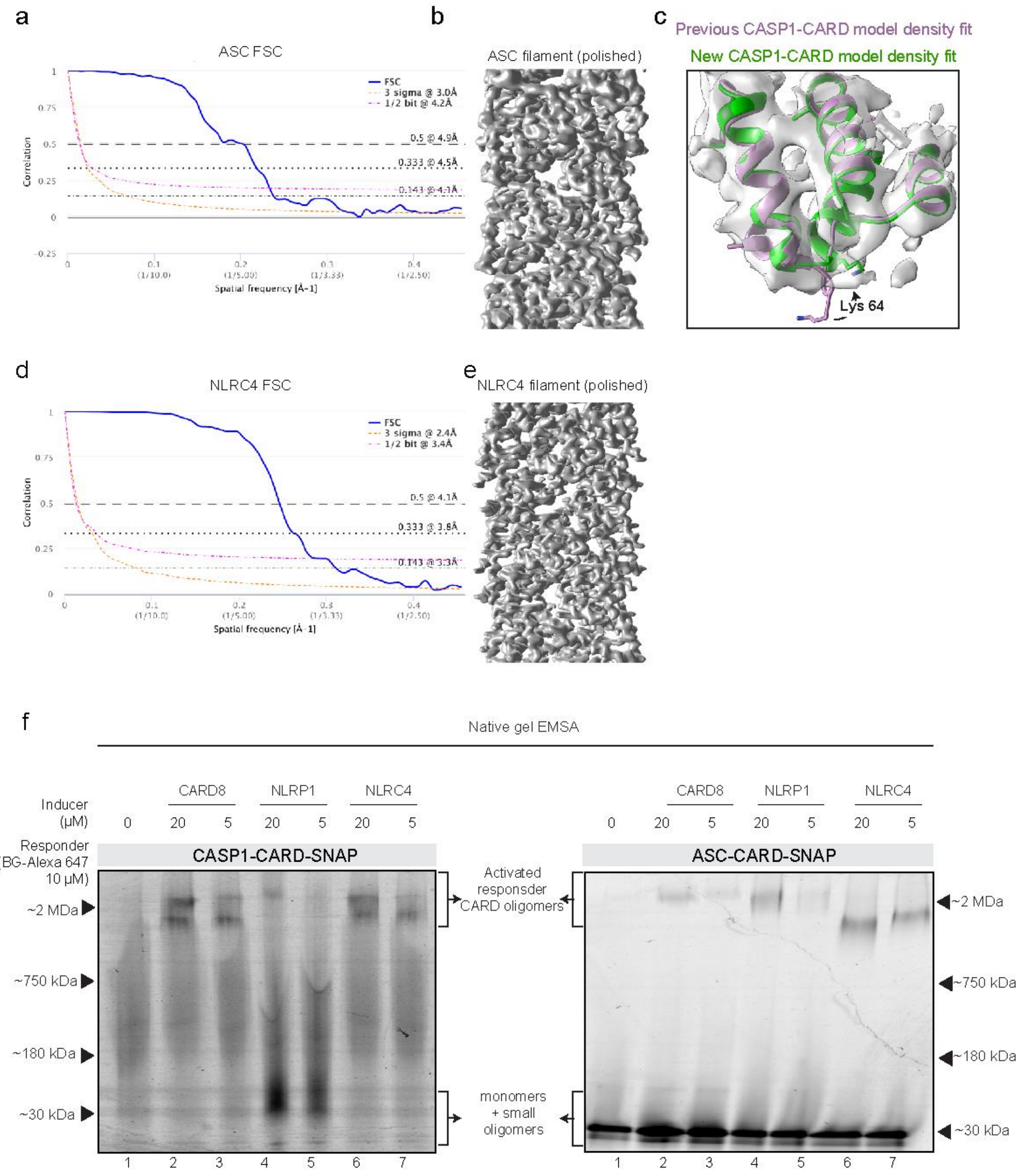

a. FSC plot of final, post-refinement ASC-CARD filament density (EMD-9947).
b. Post-refinement 3D density of ASC-CARD filament.
c. Refined fitting of CASP1-CARD density (EMD-3241). Purple is the original PDB fitting (PDB-5FNA). Green is our new, refined fitting based on the newly acquired, homologous CARD8-CARD structure. A shift of amino acid register was identified, supported by Lys64 density assignment.
d. FSC plot of final, post-refinement NLRC4-CARD filament density (EMD-9946).
e. Post-refinement 3D density of NLRC4-CARD filament.
f. Left: native gel shift assay of recombinant CASP1-CARD-SNAP oligomerization induced by purified CARD8-CARD, NLRP1-CARD and NLRC4-CARD. Right: native gel shift assay of ASC-CARD-SNAP oligomerization induced by purified CARD8-CARD, NLRP1-CARD and NLRC4-CARD.

Supplementary Figure 7. Full-length NLRP1- and CARD8-mCherry filaments observed in Talabostat-treated 293T cells.

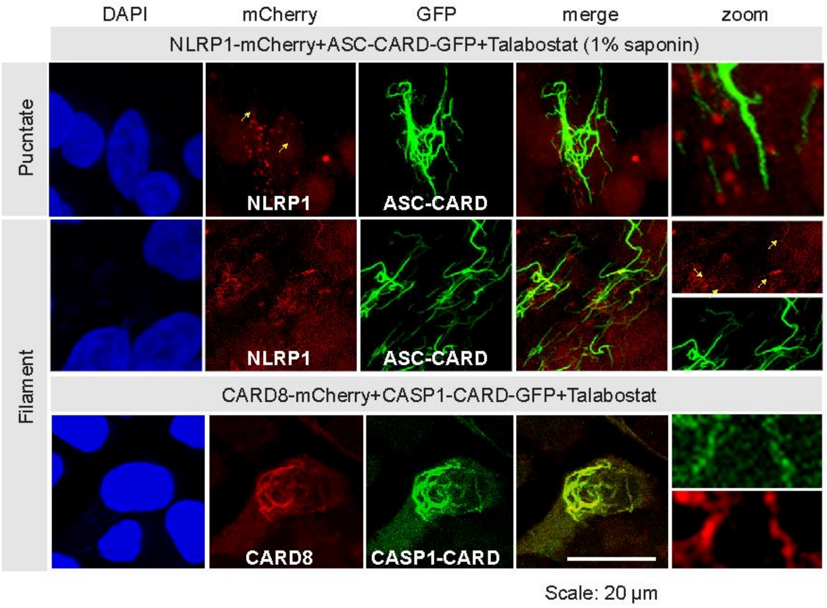

Supplementary Figure 8. Support data of NLRP1 and CARD8 inflammasome assembly in mammalian cells

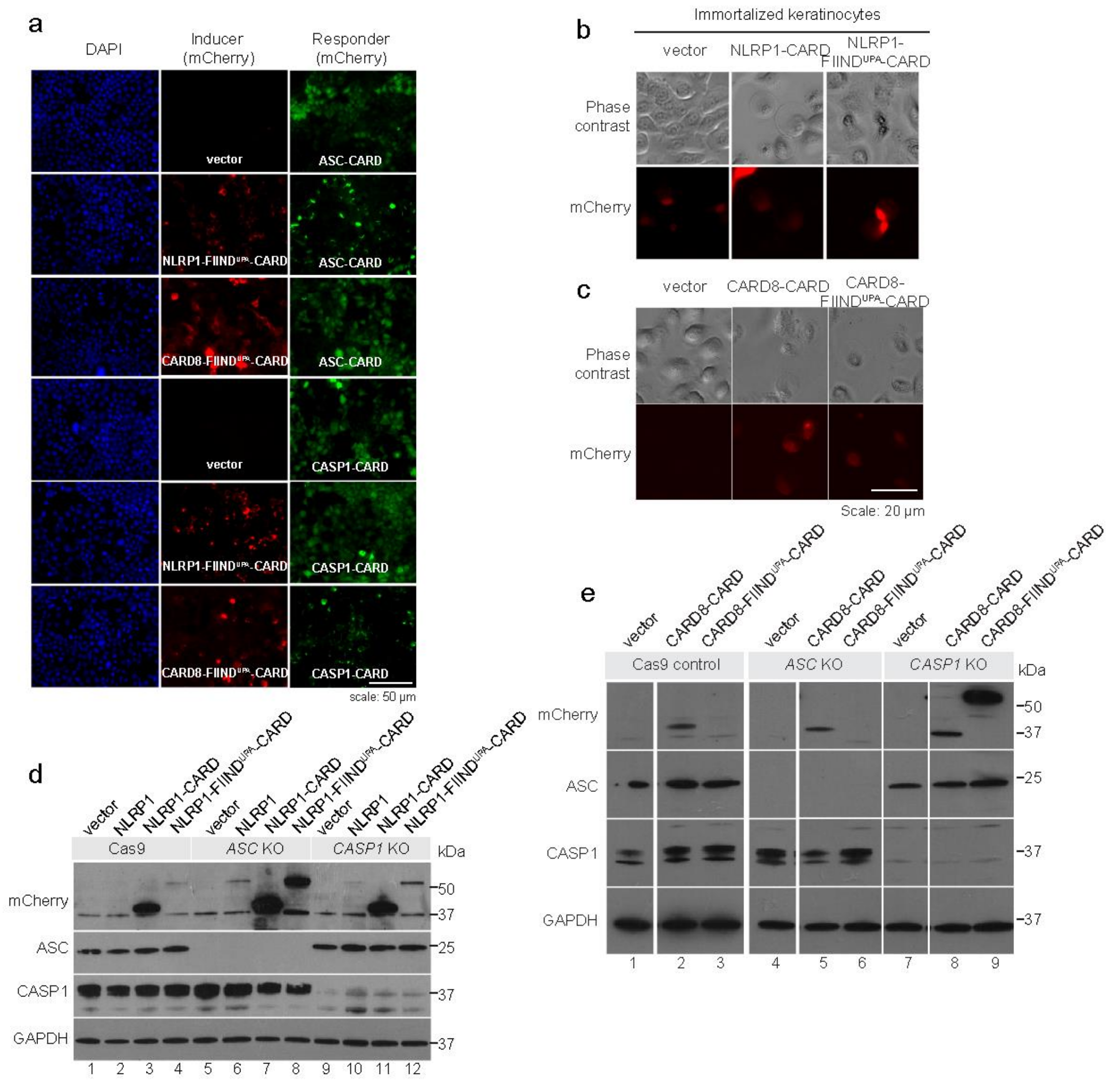

a. Representative images of 293T-ASC-CARD- and CASP1-CARD-GFP responder cells transfected with mCherry-tagged NLRP1/CARD8-FIIND^{UPA}-CARD or NLRP1/CARD8-CARD.

b. Representative images of wild-type immortalized human keratinocytes transfected with mCherry-tagged NLRP1-CARD, NLRP1-FIND^{UPA}-CARD.

c. Representative images of wild-type immortalized human keratinocytes transfected with mCherry-tagged CARD8-CARD and CARD8-FIND^{UPA}-CARD.

d. Western blot of mCherry-tagged NLRP1 constructs in control, *ASC* and *CASP1* KO keratinocytes.

e. Western blot of mCherry-tagged CARD8 constructs in control, *ASC* and *CASP1* KO keratinocytes.

Supplementary Figure 9. Only one end of the NLRP1-CARD and CARD-CARD filament is capable of seeding ASC-CARD or CASP1-CARD

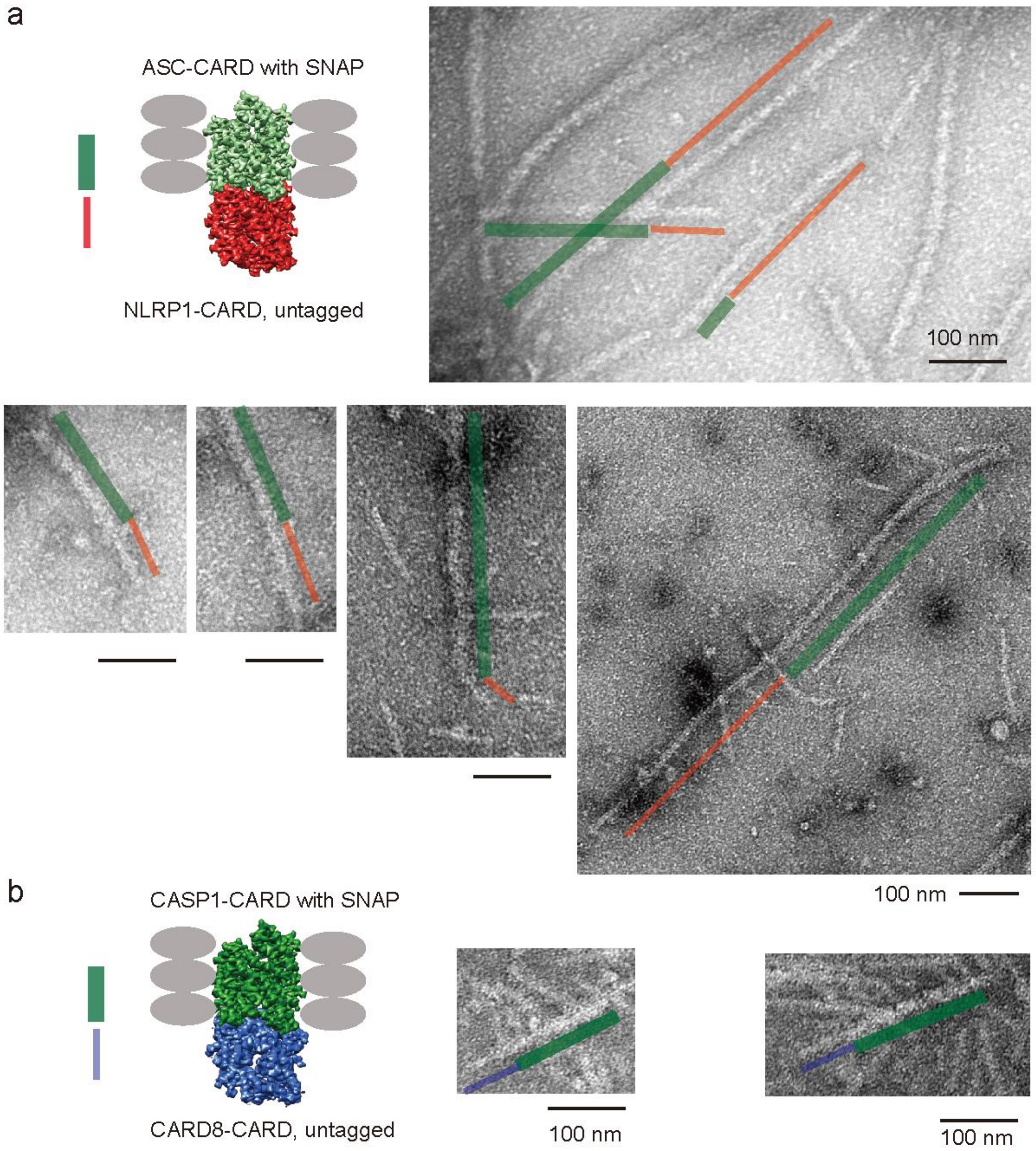

a. Negative stain EM images of ASC-CARD tagged with non-cleavage SNAP tags seeded by naked NLRP1-CARD filament segments.
b. Negative stain EM images of CASP1-CARD tagged with non-cleavage SNAP tags seeded by naked CARD8-CARD filament segments.

Supplementary Figure 10. Proposed structural interpretation of NLRC4 inflammasome assembly

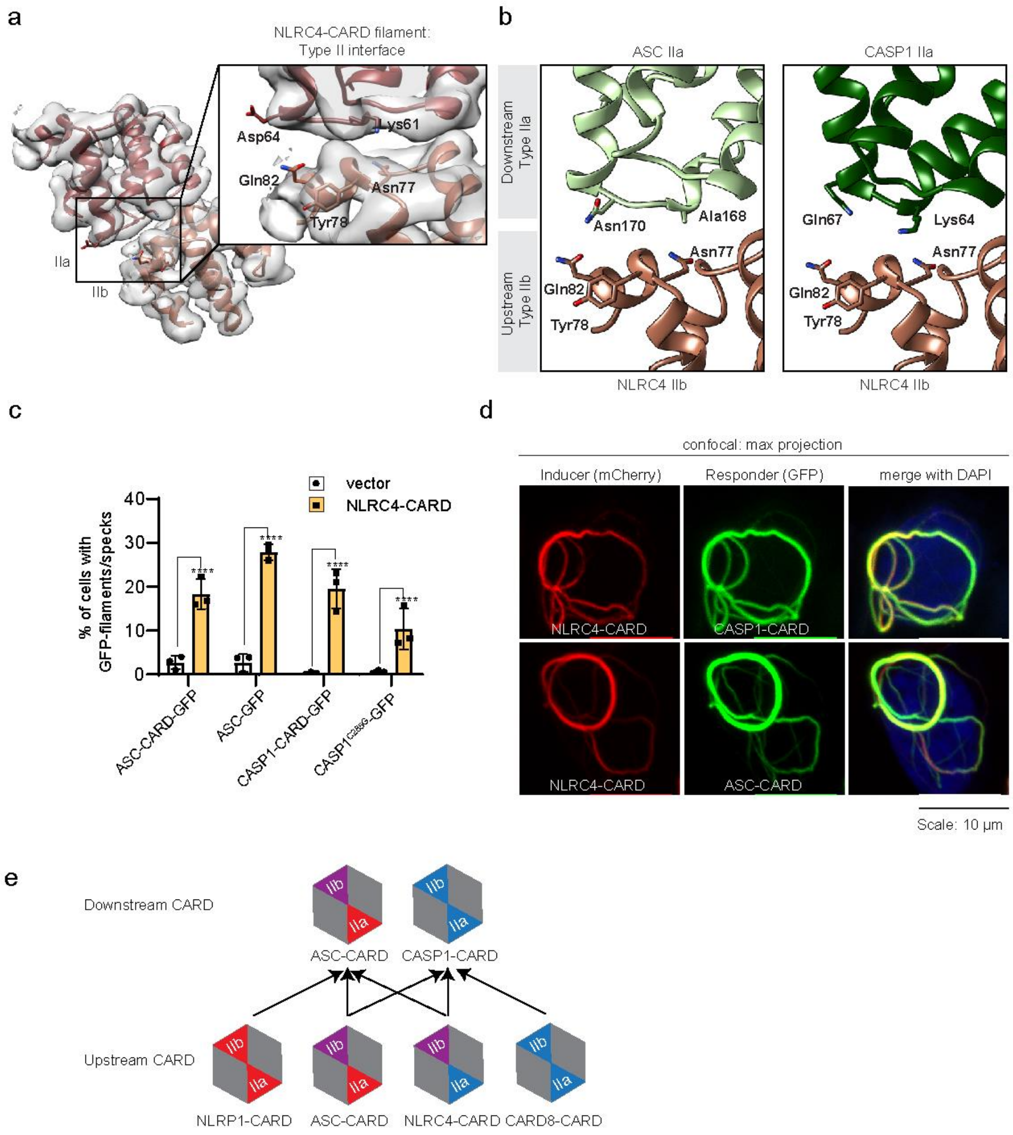

a. Atomic model and directly observed density map at Type II interface of human NLRC4-CARD filaments (PDB-6K8J, EMD-9946).
b. Type II junction interactions between NLRC4-CARD and ASC-CARD (left), and between NLRC4-CARD and CASP1-CARD (right).
c. Percentage of filament/speck formation in 293T-ASC-CARD-GFP, ASC-GFP, CASP1-CARD-GFP and CASP1^{C285G}-GFP responder cells transfected with NLRC4-CARD-mCherry. P-value was calculated with One-way ANOVA, n=3 transfections.
d. Confocal microscopy images of NLRC4-CARD-mCherry filaments with CASP1-CARD-GFP and ASC-CARD-GFP filaments in 293T cells.
e. Summary of unidirectional CARD-CARD interactions in human NLRP1, CARD8 and NLRC4 inflammasome complexes.

Supplementary Figure 11. Power spectrum analysis of filamentous samples.

NLRP1-CARD filament Power Spectrum
Theoretical (simulated 1 Å, top Vs. real 2D class image, bottom)

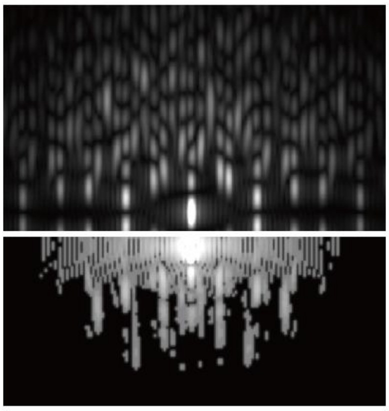

CARD8-CARD filament Power Spectrum
Theoretical (simulated 1 Å, top Vs. real 2D class image, bottom)

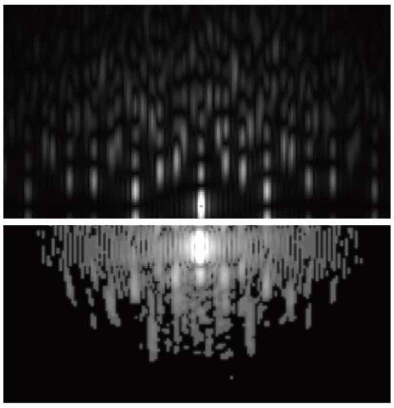

ASC-CARD filament Power Spectrum
Theoretical (simulated 1 Å, top Vs. real 2D class image, bottom)

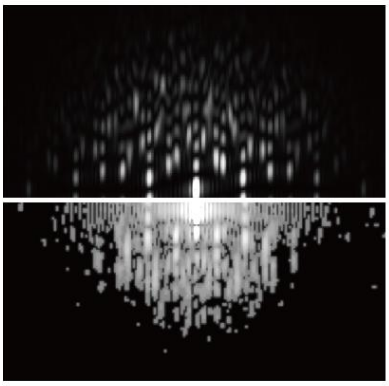

NLRC4-CARD filament Power Spectrum
Theoretical (simulated 1 Å, top Vs. real 2D class image, bottom)

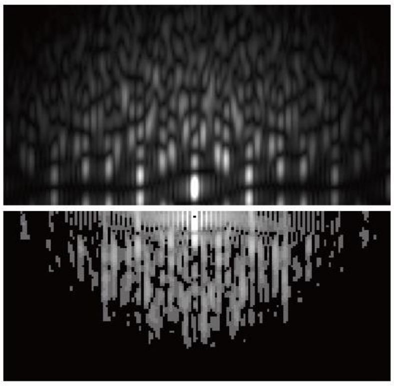

NLRP1-FIIND^{UPA}-CARD filament Power Spectrum
Theoretical (simulated 20 Å, top Vs. real 2D class image, bottom)

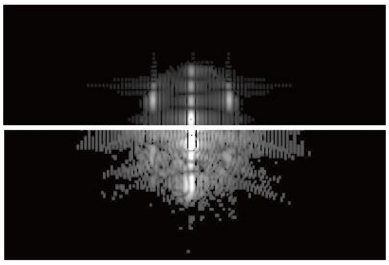

Supplementary Table 1. Data Collection, map and model refinement, validation

	ASC	NLRC4	CARD8	NLRP1
**Data Collection**				
Microscope	Titan Krios	Titan Krios	Titan Krios	Titan Krios
Voltage (kV)	300	300	300	300
Detector	K2	K2	K2	K2
Pixel size (Å)	1.1	1.1	1.1	1.1
Defocus range (μm)	-1,-2	-1,-2	-1,-2	-1,-2
Electron Dose (e^-/A^2)	40	40	40	40
**Helical Reconstruction**				
Software	RELION 3.0	RELION 3.0	RELION 3.0	RELION 3.0
Segment length (Å)	220	220	264	264
Particles	877408	382715	1201108	422388
Helical rise (Å)	5.27	5.17	5.409	5.36
Helical rotation (°)	-100.614	-100.55	-99.16	-100.821
Resolution (Å)	4.1	3.3	3.7	3.7
**Coordinate Refinement (mid-segment 12mer)**				
Software	Phenix	Phenix	Phenix	Phenix
Rwork	0.211	0.2571	0.2530	0.2548
Rfree	0.2208	0.3057	0.3017	0.2846
**Model**				
Number of residues	996	1020	1044	1008
B-factor overall	206	249	276	360
R.M.S. deviation				
Bond length (Å)	0.003	0.002	0.002	0.005
Bond angle (°)	0.670	0.364	0.423	0.767
**Validation**				
Molprobity clashscore	6.92	0.24	5.41	2.06
Rotamer Outliers (%)	1.3	0	0	1.5
C_β deviation (%)	0	0	0	0
**Ramachandran plot**				
Favored (%)	92.5	98.1	100	96.5
Allowed (%)	6.2	1.9	0	3.5
Outliers (%)	0	0	0	0
