# Supplementary material for: Structural basis for distinct inflammasome complex assembly by human NLRP1 and CARD8
Source: Nat Commun. 2021 Jan 8;12:188. doi: 10.1038/s41467-020-20319-5 (PMC7794362; doi:10.1038/s41467-020-20319-5)
Supplement: Supplementary file 3 — Reporting Summary [file 41467_2020_20319_MOESM3_ESM.pdf]

nature research

Corresponding author(s): Bin Wu

Last updated by author(s): Nov 14, 2020

Reporting Summary

Nature Research wishes to improve the reproducibility of the work that we publish. This form provides structure for consistency and transparency in reporting. For further information on Nature Research policies, see Authors & Referees and the Editorial Policy Checklist .

Statistics

For all statistical analyses, confirm that the following items are present in the figure legend, table legend, main text, or Methods section.

n/a	Confirmed	
☐	☒	The exact sample size (*n*) for each experimental group/condition, given as a discrete number and unit of measurement
☐	☒	A statement on whether measurements were taken from distinct samples or whether the same sample was measured repeatedly
☐	☒	The statistical test(s) used AND whether they are one- or two-sided<br>*Only common tests should be described solely by name; describe more complex techniques in the Methods section.*
☒	☐	A description of all covariates tested
☒	☐	A description of any assumptions or corrections, such as tests of normality and adjustment for multiple comparisons
☐	☒	A full description of the statistical parameters including central tendency (e.g. means) or other basic estimates (e.g. regression coefficient) AND variation (e.g. standard deviation) or associated estimates of uncertainty (e.g. confidence intervals)
☐	☒	For null hypothesis testing, the test statistic (e.g. *F*, *t*, *r*) with confidence intervals, effect sizes, degrees of freedom and *P* value noted<br>*Give P values as exact values whenever suitable.*
☒	☐	For Bayesian analysis, information on the choice of priors and Markov chain Monte Carlo settings
☒	☐	For hierarchical and complex designs, identification of the appropriate level for tests and full reporting of outcomes
☒	☐	Estimates of effect sizes (e.g. Cohen's *d*, Pearson's *r*), indicating how they were calculated

Our web collection on statistics for biologists contains articles on many of the points above.

Software and code

Policy information about availability of computer code

Data collection	Thermofisher Scientific EPU 2.4, SoftWorX 6.5.2
Data analysis	MotionCorr2, RELION 2.0, RELION 3.0beta, RELION 3.0.8, Coot 0.8.9, UCSF Chimera 1.13, Phenix 1.17, cisTEM1.0beta, Imaris version 9.3.0, Fiji (2017 May package) is used for 2D image analysis. ChimeraX 1.1 is used for preparing 3D density images. Illustrator 22 was used for preparing figures.

For manuscripts utilizing custom algorithms or software that are central to the research but not yet described in published literature, software must be made available to editors/reviewers. We strongly encourage code deposition in a community repository (e.g. GitHub). See the Nature Research guidelines for submitting code & software for further information.

Data

Policy information about availability of data

All manuscripts must include a data availability statement. This statement should provide the following information, where applicable:
- Accession codes, unique identifiers, or web links for publicly available datasets
- A list of figures that have associated raw data
- A description of any restrictions on data availability

NLRC4-CARD filaments (PDB-6K8J, https://www.rcsb.org/structure/6K8J) and (EMD-9946, https://www.emdataresource.org/EMD-9946);ASC-CARD filaments (PDB-6K99, https://www.rcsb.org/structure/6K99) and (EMD-9947, https://www.emdataresource.org/EMD-9947); NLRP1-CARD (PDB-6K7V, https://www.rcsb.org/structure/6K7V) and (EMD-9943, https://www.emdataresource.org/EMD-9943); CARD8-CARD (PDB-6K9F, https://www.rcsb.org/structure/6K9F) and (EMD-9948, https://www.emdataresource.org/EMD-9948).

Field-specific reporting

Please select the one below that is the best fit for your research. If you are not sure, read the appropriate sections before making your selection.

- [x] Life sciences
- [ ] Behavioural & social sciences
- [ ] Ecological, evolutionary & environmental sciences

For a reference copy of the document with all sections, see nature.com/documents/nr-reporting-summary-flat.pdf

Life sciences study design

All studies must disclose on these points even when the disclosure is negative.

Sample size	Number of images and particles for structural analysis or image analysis were explicitly described in methods or figure legend. In general, three repeats are conducted for biochemical experiments (for example, luciferase assay, ELISA, EMSA, etc), since three is required for basic statistical analysis. For samples/experiments that will be used for final figures, we will do one addition experiment. Negative stain EM experiments were independently repeated for at least 5 times, since protein quality varies and there are additional variations in staining, blotting, machine conditions, etc. When analyzing spatial properties (for example, diameter of filament) of particular objects in EM images, we choose more than ten most representative objects from different images, and at least from two independent experiments, with better contrast. We choose these values in order to avoid bias from a single experiment, due to potential errors in EM optics (so pixel size may be off), sample contaminations, etc.
Data exclusions	Poor cryo-EM images, which were determined to be too noisy, were excluded from further 2D and 3D density building based on standard statistical analysis in RELION 3.0 software.
Replication	Repeated efforts were attempted to obtain refolded CARD-domain filaments. Once protocol was optimized, the results were highly reproducible. Both NLRP1 and CARD8 CARD thin filament were performed more than 10 times using both negative stain EM and cryo-EM, the results were highly reproducible and consistent. NLRP1-UPA-CARD filament negative stain EM experiment was repeated 21 times from 2018 and 2019, 5 were successful, seeing the same ordered thick filament, and generated enough filament images that could be used for low resolution structural determination. NLRP1-UPA-CARD constructs have very high tendency to precipitate. CARD8-UPA-CARD filament negative stain EM experiment was repeated 11 times in 2019 and 2020, before linker modification, 2 of the attempts produced images with good contrast, seeing ordered thick filament. After linker optimization, CARD8-UPA-CARD filaments were obtained 4 times out of 5 attempts in mid 2020. Co-seeding experiments were attempted 2 times, both were successful.
Randomization	We designed biochemical experiments after solving the structures, so we already have prior knowledge of the roles played by the amino acids. Although we tried to cover the entire surface of the domain surface during biochemical validation, indeed we intentionally selected specific residues for positive and negative control purposes.
Blinding	Blank density model with random Gassian noise was used as the starting model for non-guided helical reconstruction during cyro-EM model building. We did label samples in mutation studies with symbols, and not revealing the identity of the actual mutant to the student who was conducting the ELISA and luciferase experiments. EM experiments are difficult to include blinding practice, since we need to know the expected appearance of the objects to perform the particle and segment picking.

Reporting for specific materials, systems and methods

We require information from authors about some types of materials, experimental systems and methods used in many studies. Here, indicate whether each material, system or method listed is relevant to your study. If you are not sure if a list item applies to your research, read the appropriate section before selecting a response.

Materials & experimental systems

n/a	Involved in the study	
[ ]	[x]	Antibodies
[ ]	[x]	Eukaryotic cell lines
[x]	[ ]	Palaeontology
[x]	[ ]	Animals and other organisms
[x]	[ ]	Human research participants
[x]	[ ]	Clinical data

Methods

n/a	Involved in the study	
[x]	[ ]	ChIP-seq
[x]	[ ]	Flow cytometry
[x]	[ ]	MRI-based neuroimaging

Antibodies

Antibodies used	HA tag (Santa Cruz Biotechnology, #sc-805), GAPDH (Santa Cruz Biotechnology, #sc-47724), ASC (Adipogen, #AL-177), CASP1 (Santa Cruz Biotechnology, #sc-622), IL1B (R&D systems, #AF-201), FLAG (SigmaAldrich, #F3165), GFP (Abcam, #ab290). IL-1B measurements were carried out with human IL-1B ELISA kit (BD, #557953). Dilution factor were included in the method section.
Validation	Validated by commercial providers, according to statement on the suppliers' website. In addition, we have validated these antibodies (lots may vary) in previous studies. HA tag, ASC, CASP1, FLAG, GFP antibodies were internally validated by our own

recombinant protein in previous western blot or imaging experiments. IL1B antibody was validated according to supplier's instruction. It was claimed that 'Western blot shows lysates of THP-1 human acute monocytic leukemia cell line untreated (-) or treated (+) with 200 nM PMA for 24 hours and 10 µg/mL LPS and 3 hours. PVDF membrane was probed with 0.1 µg/mL of Goat Anti-Human IL-1 beta /IL-1F2 Antigen Affinity-purified Polyclonal Antibody (Catalog # AF-201-NA) followed by HRP-conjugated Anti-Goat IgG Secondary Antibody (Catalog # HAF017). A specific band was detected for IL-1 beta /IL-1F2 at approximately 36 kDa (as indicated). This experiment was conducted under reducing conditions and using Immunoblot Buffer Group 1.' and IL-1B ELISA kit were calibrated with recombinant IL1B, as claimed by manufacturer. GAPDH antibody was also validated by manufacturer using recombinant protein.

Eukaryotic cell lines

Policy information about cell lines

Cell line source(s)	HEK293Ts (ATCC #CRL-3216) were obtained from commercial sources and cultured according to the suppliers' protocols. Immortalized human keratinocytes (N/TERT-1) were a kind gift from H. Reinwald (MTA).
Authentication	Use according to suggested protocol. Expression level of Inflammasome components fit with previous studies. Expression profile of inflammasome related components, including NLRP1, CARD8, ASC, CASP1, IL1b, etc were verified in this study. No further cell line validation experiments were performed.
Mycoplasma contamination	All cell lines underwent routine Mycoplasma testing with Lonza MycoAlert (Lonza #LT07-118). All cell lines that eventually used for our experiments tested negative for mycoplasma contamination.
Commonly misidentified lines (See ICLAC register)	No commonly misidentified cell lines were used in the study.
