# Supplementary material for: Structural basis for distinct inflammasome complex assembly by human NLRP1 and CARD8
Source: Nat Commun. 2021 Jan 8;12:188. doi: 10.1038/s41467-020-20319-5 (PMC7794362; doi:10.1038/s41467-020-20319-5)

# Structural basis for distinct inflammasome complex assembly by human NLRP1 and CARD8

Gong et al., 2020

**Additional Source Data files:**

**Original blots and gels**

# Final Fig 1c-d as seen in manuscript

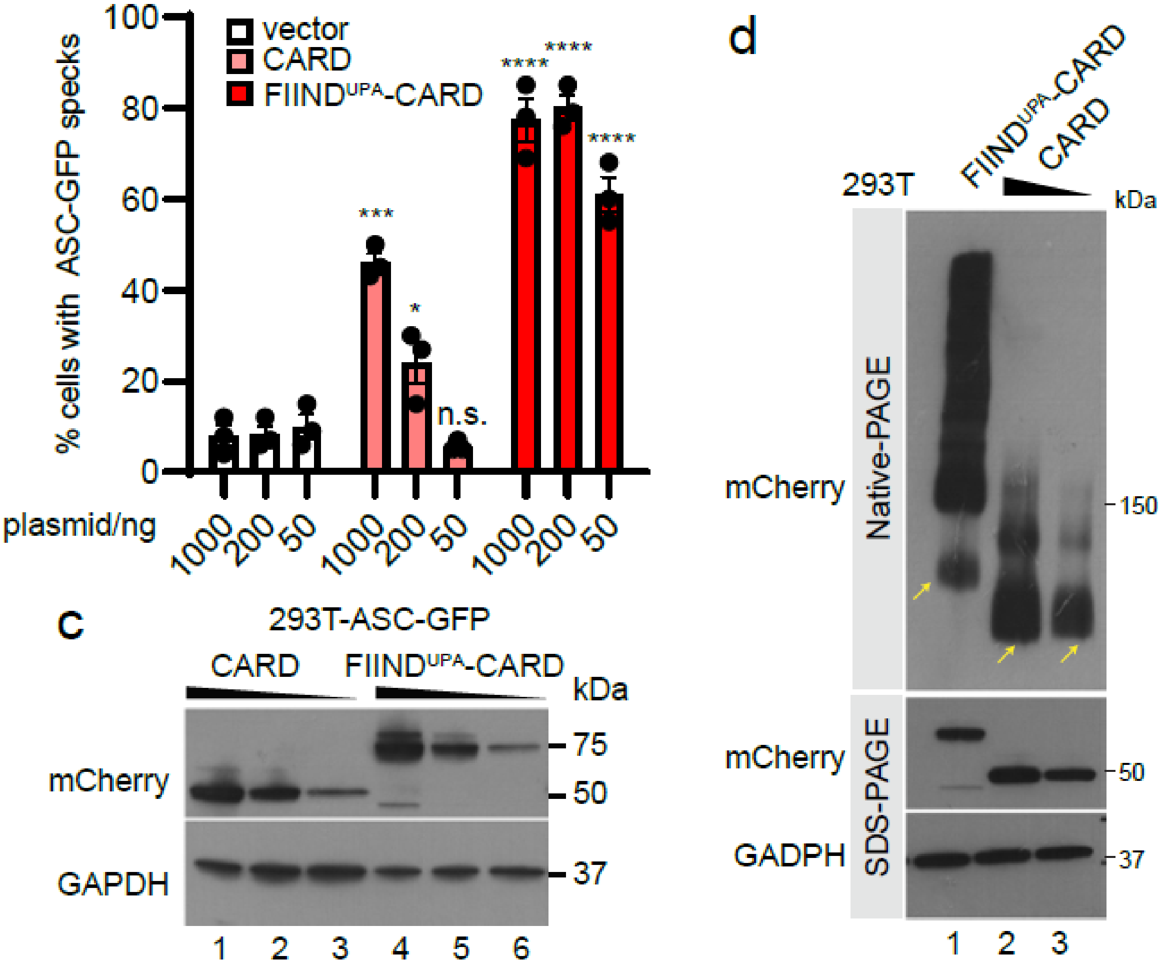

# Original blot for Fig. 1c

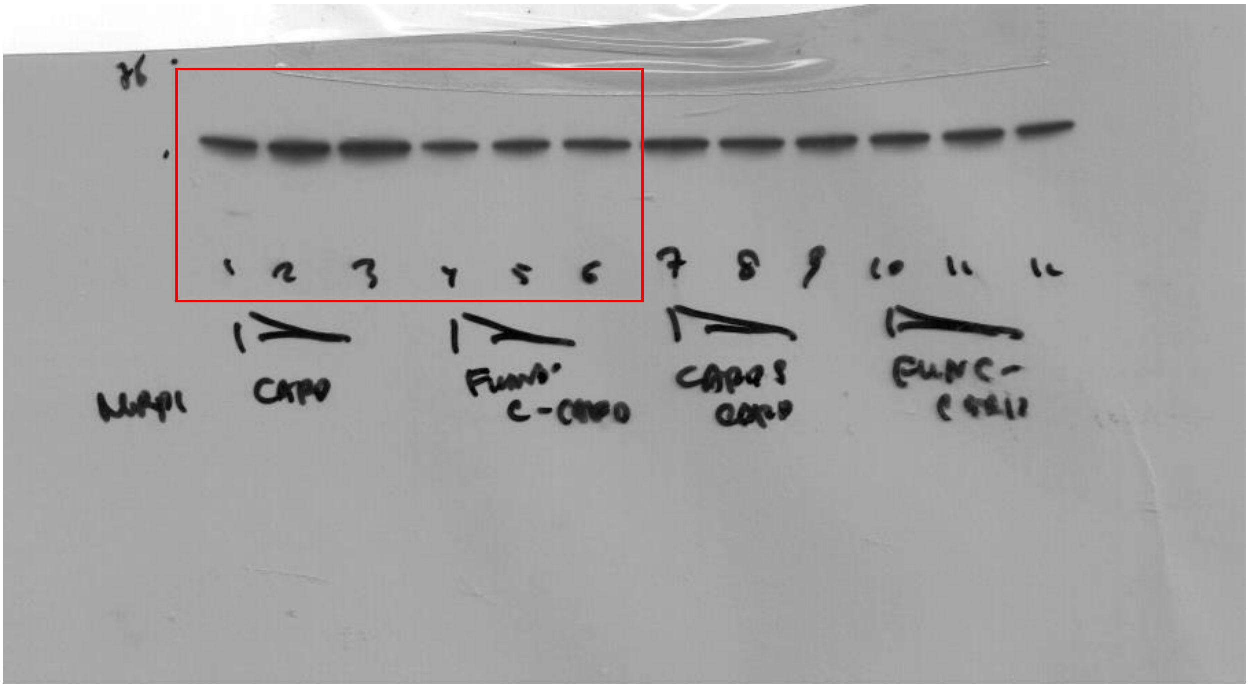

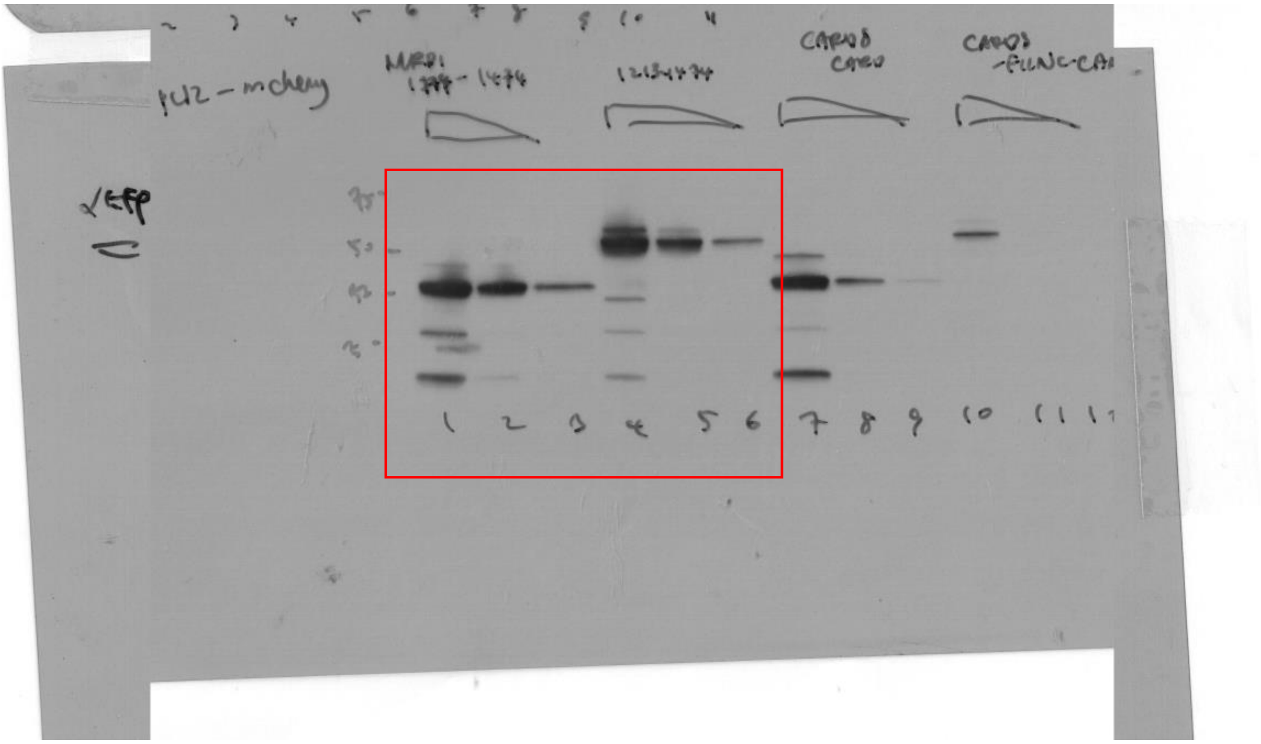

# Original Blot for Fig 1d (1 of 3)

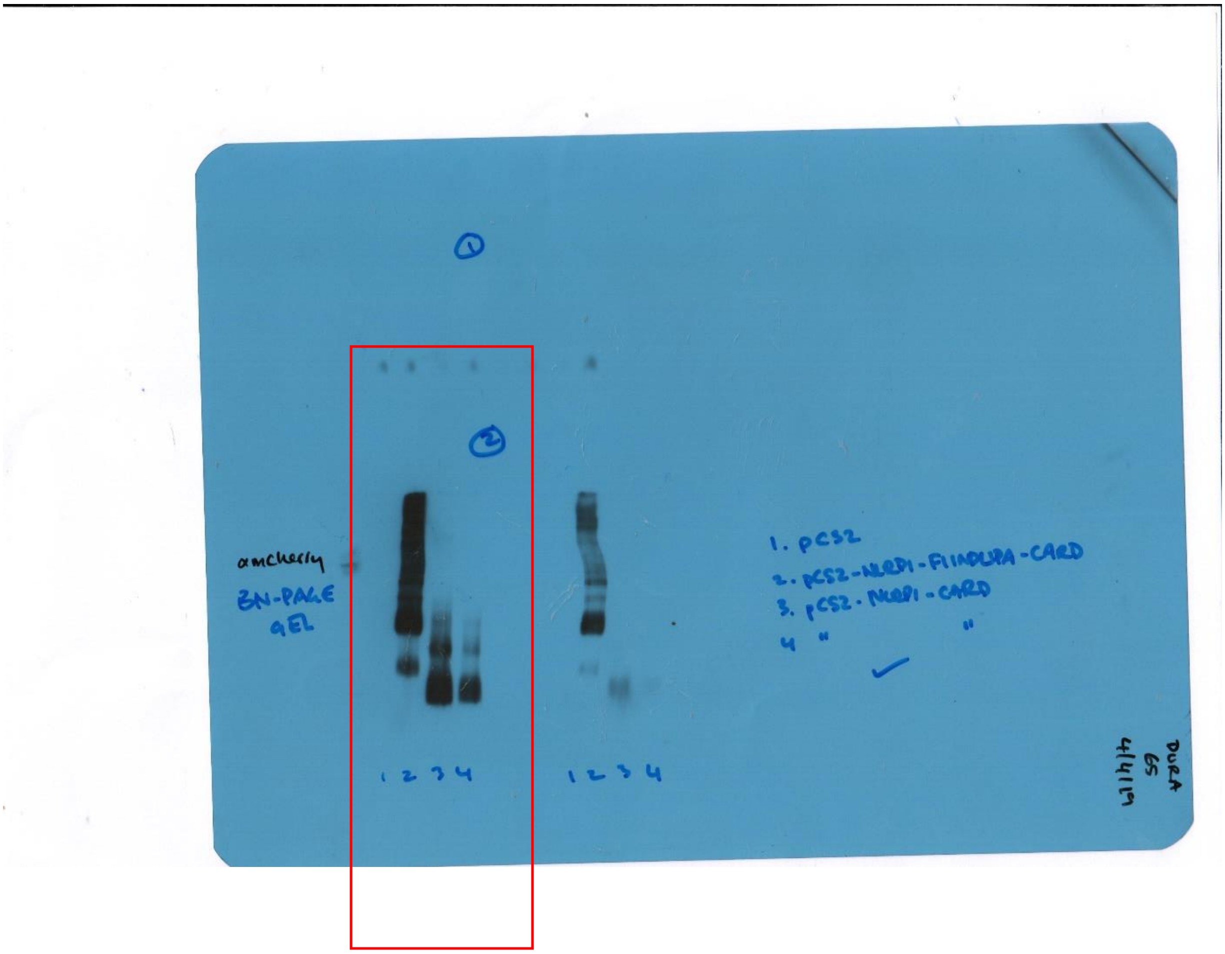

# Original Blot for Fig 1d (2 of 3)

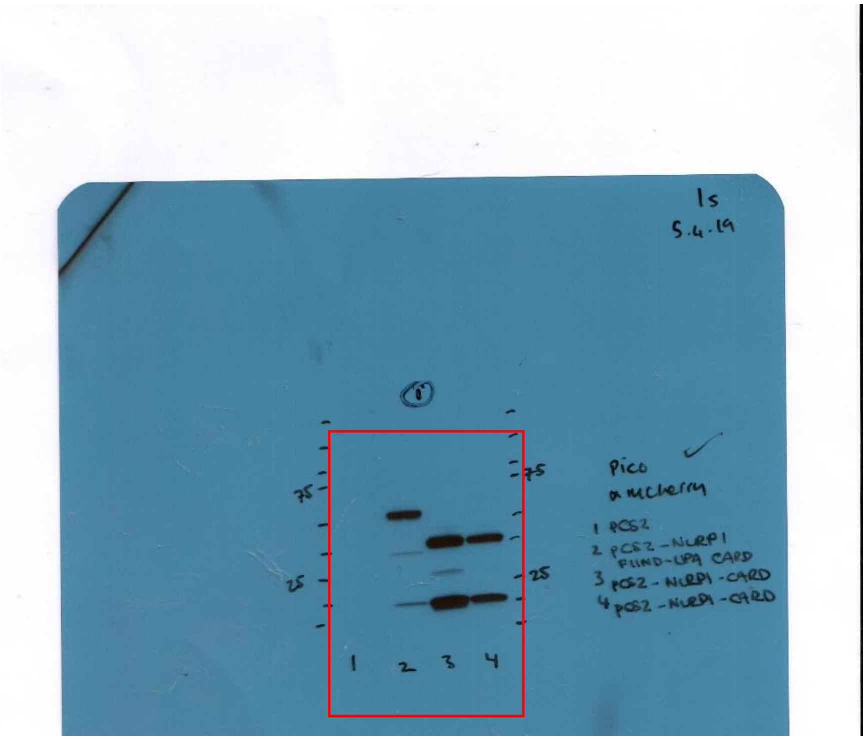

# Original Blot for Fig 1d (3 of 3)

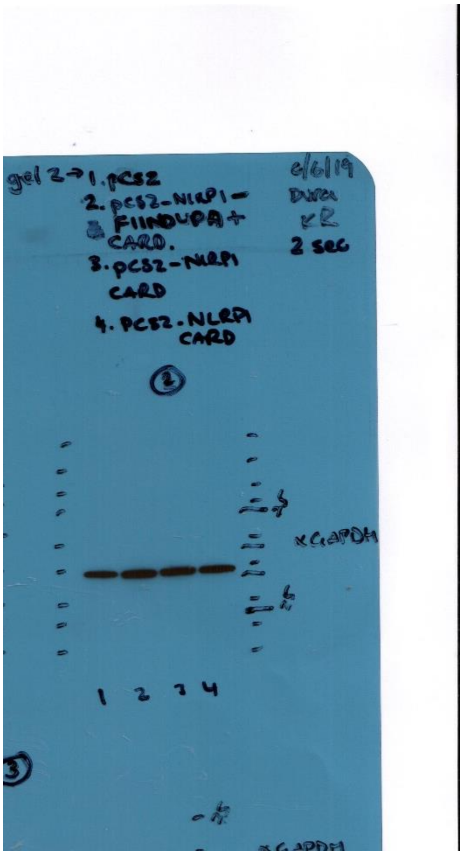

# Figure 3a as seen in manuscript

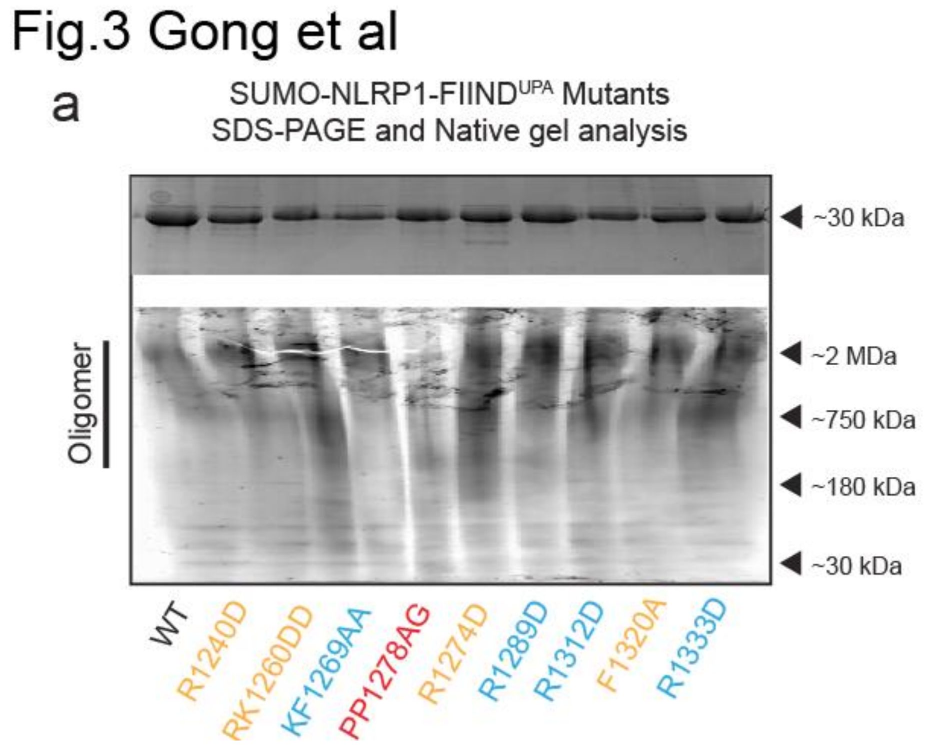

# Original SDS-PAGE images for Figure 3a

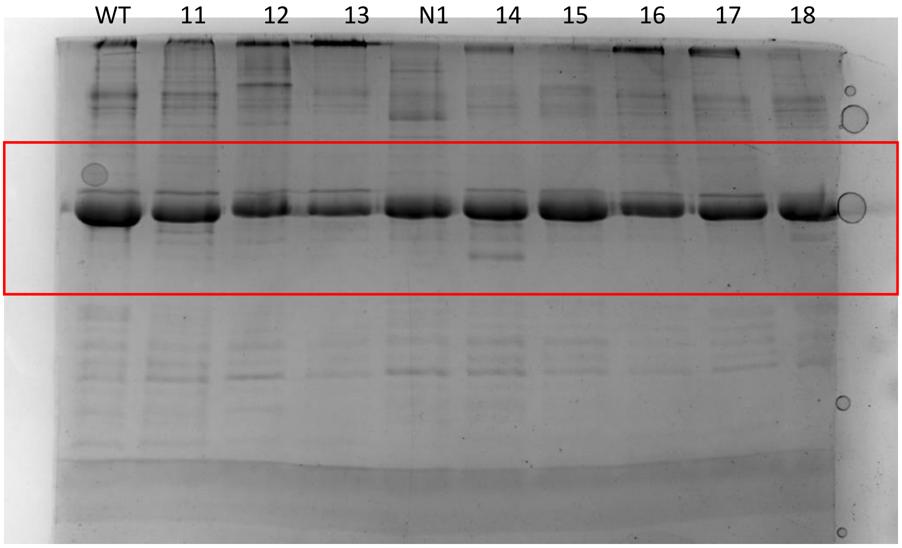


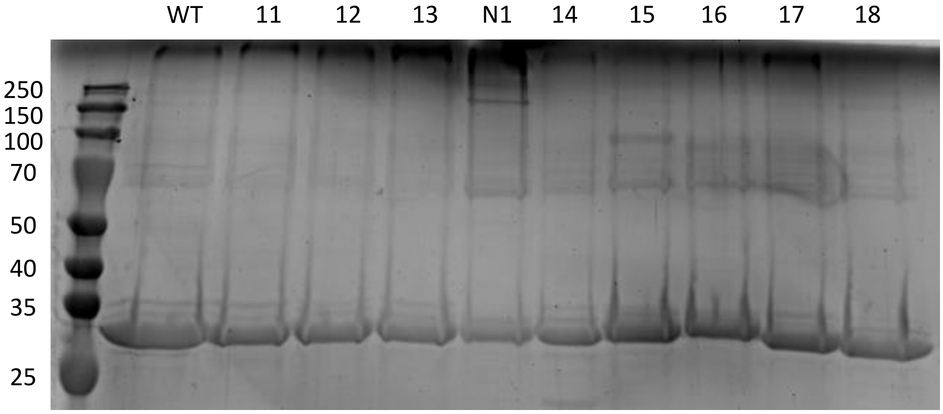

# Original native Bis-Tris image for Figure 3a

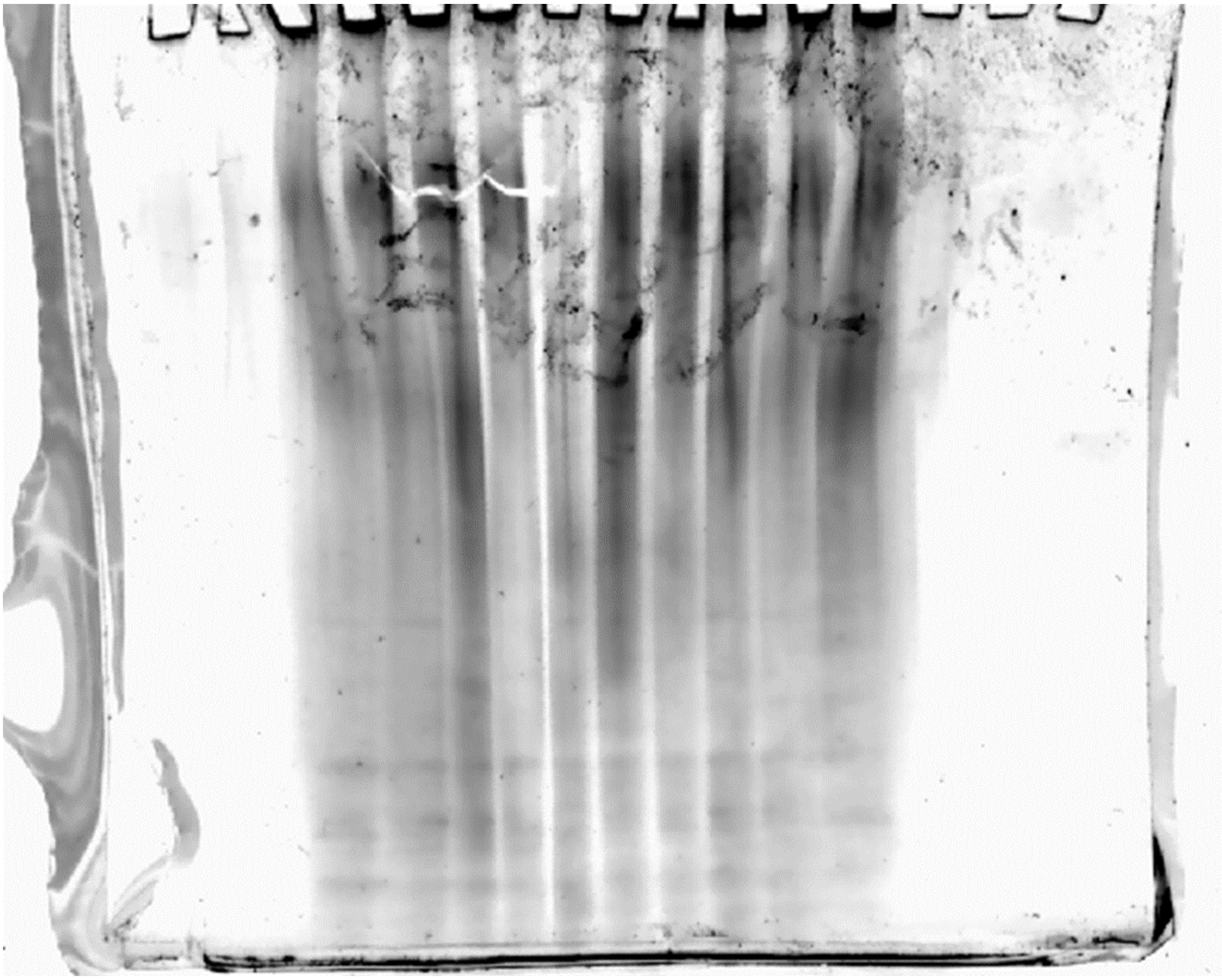

# Supplementary Figure 1d as seen in the manuscript

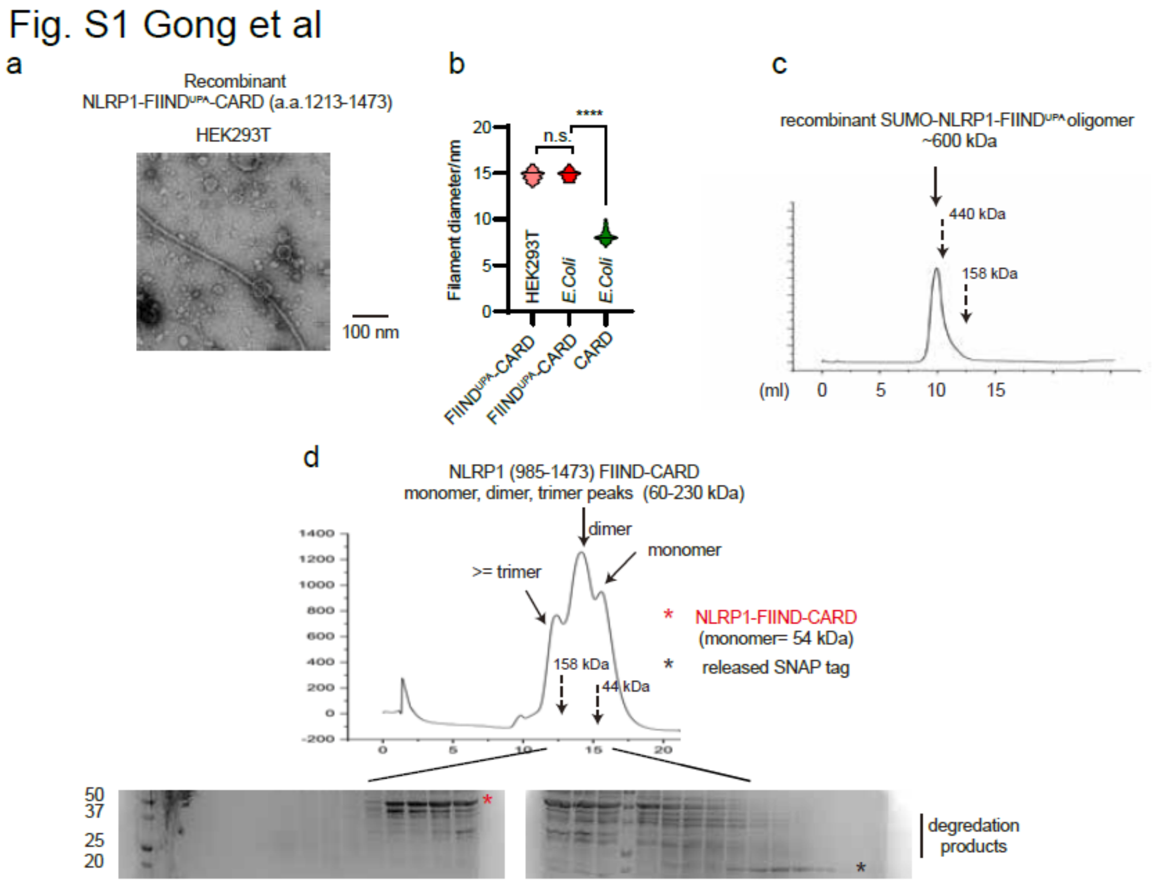

# Original SDS-PAGE images for Supplementary Figure 1d

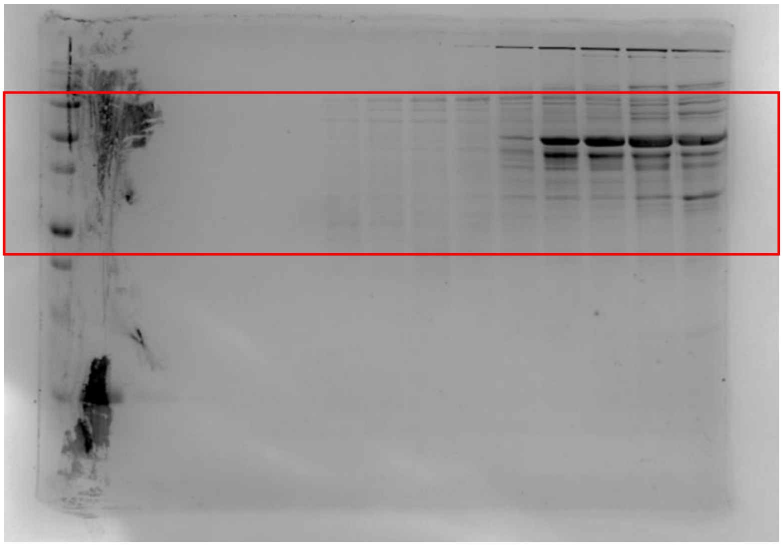

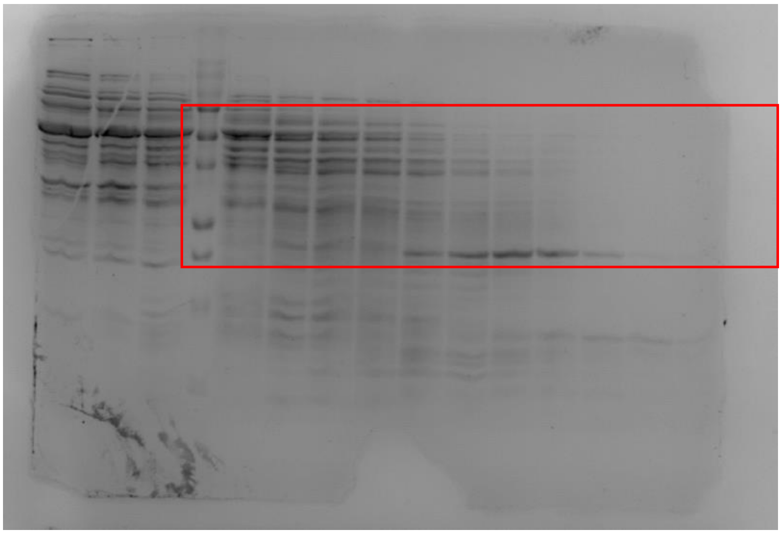

# Final Supplementary Figure 2b as seen in manuscript

Fig.S2 Gong et al

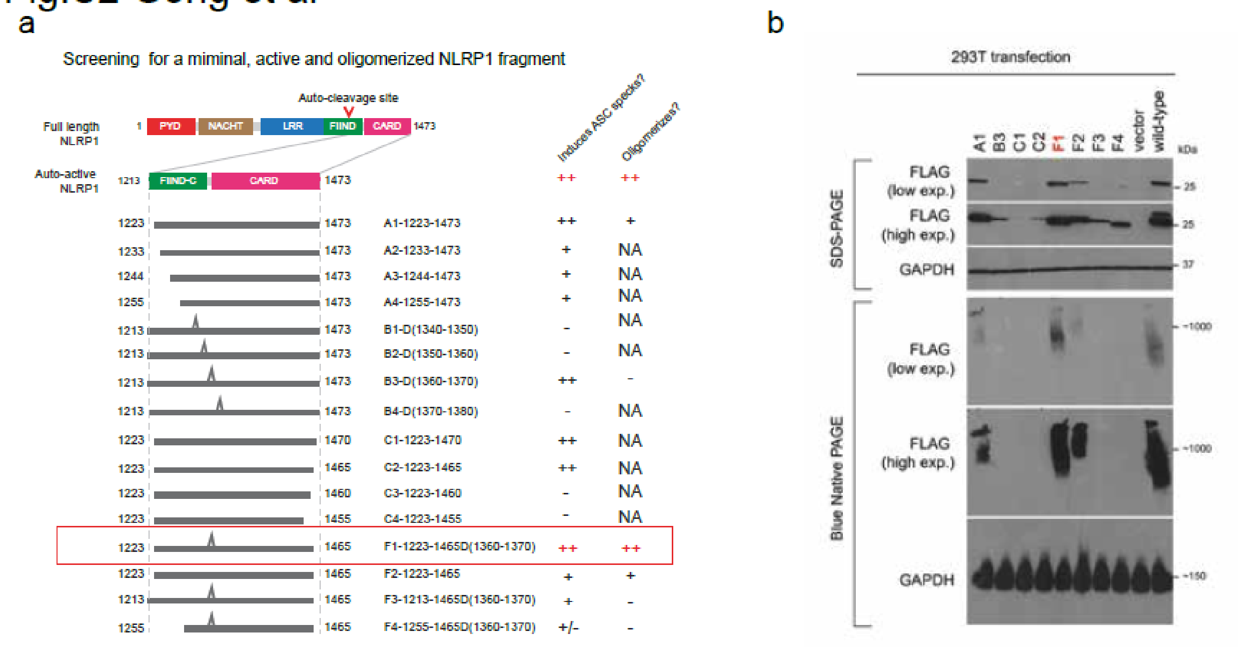

# Original Blot for Supplementary Figure 2b (1 of 3) Top panel

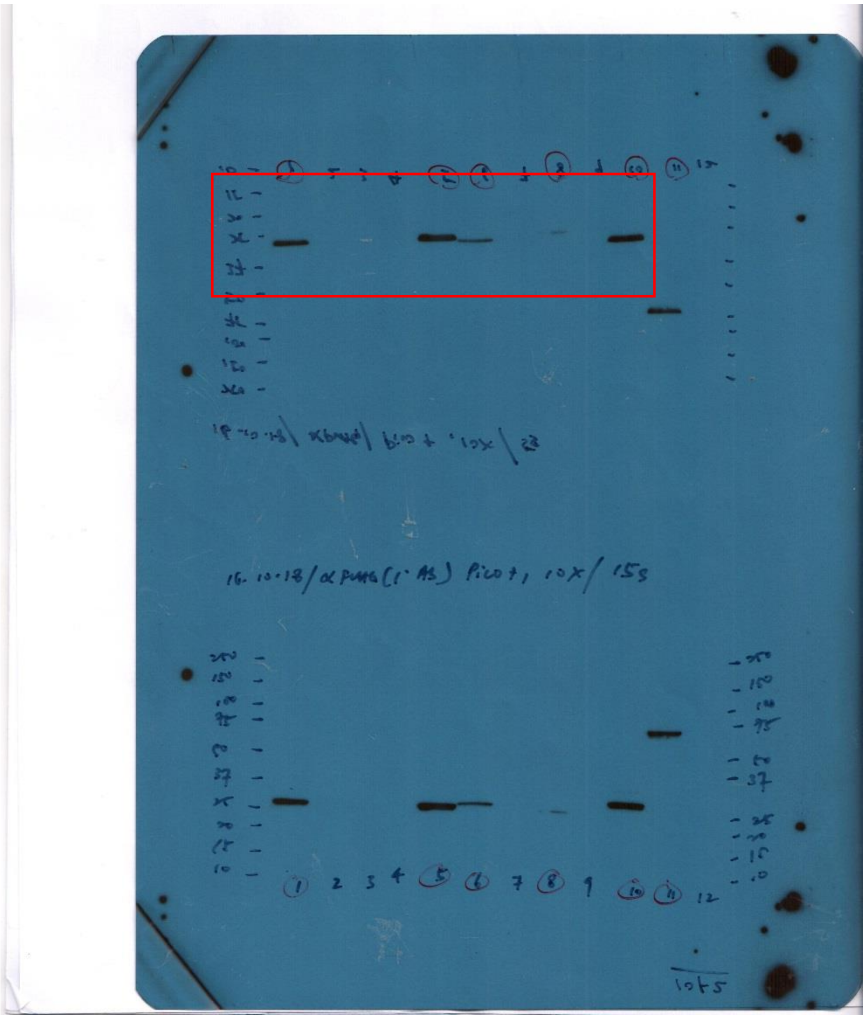

# Original Blot for Supplementary Figure 2b (2 of 3)

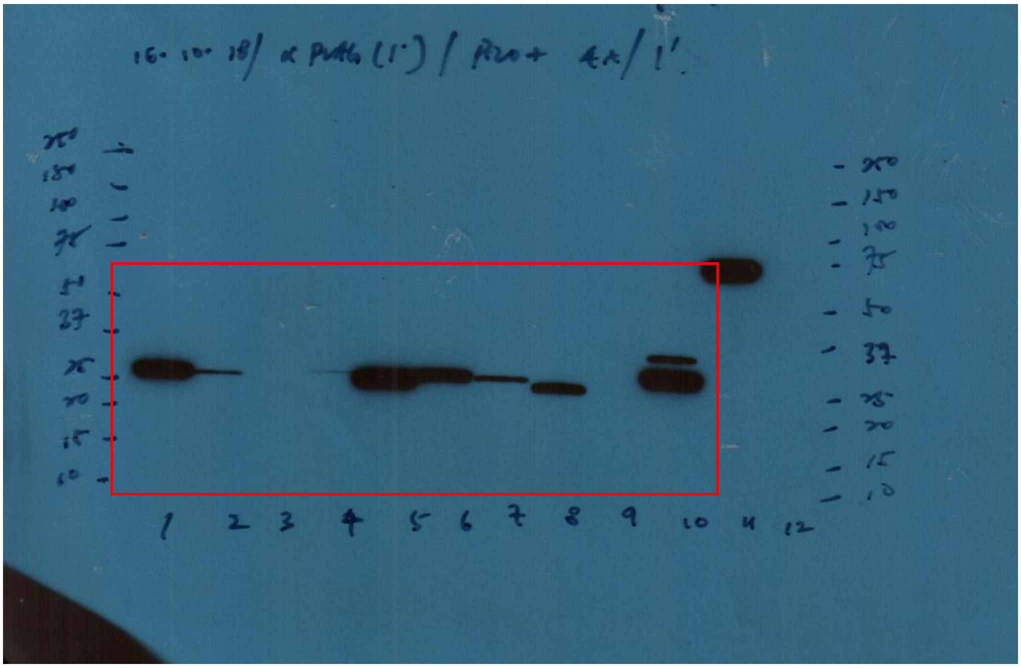

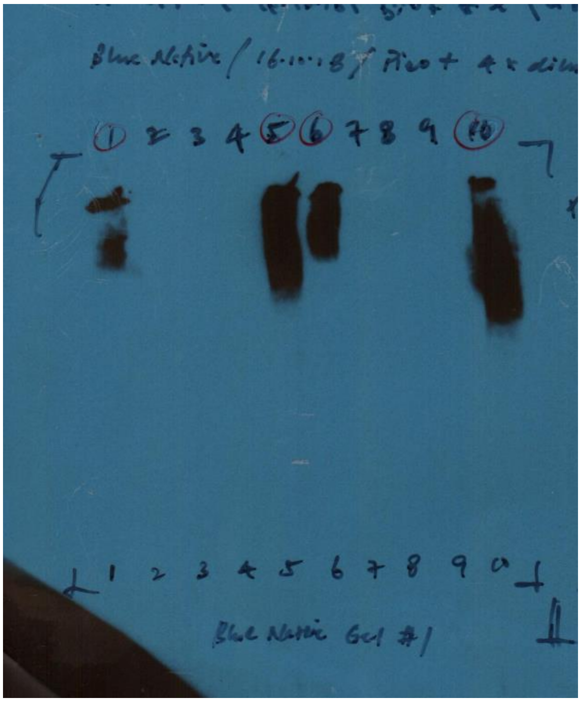

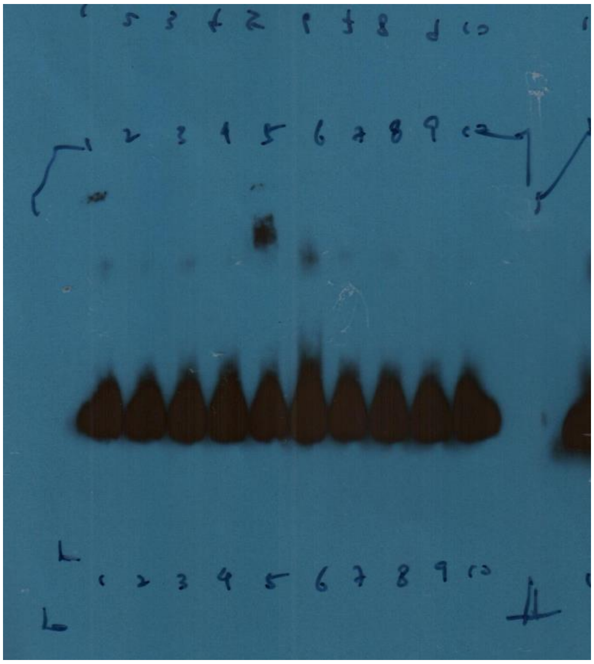

# Original Blot for Supplementary Figure 2b (3 of 3)

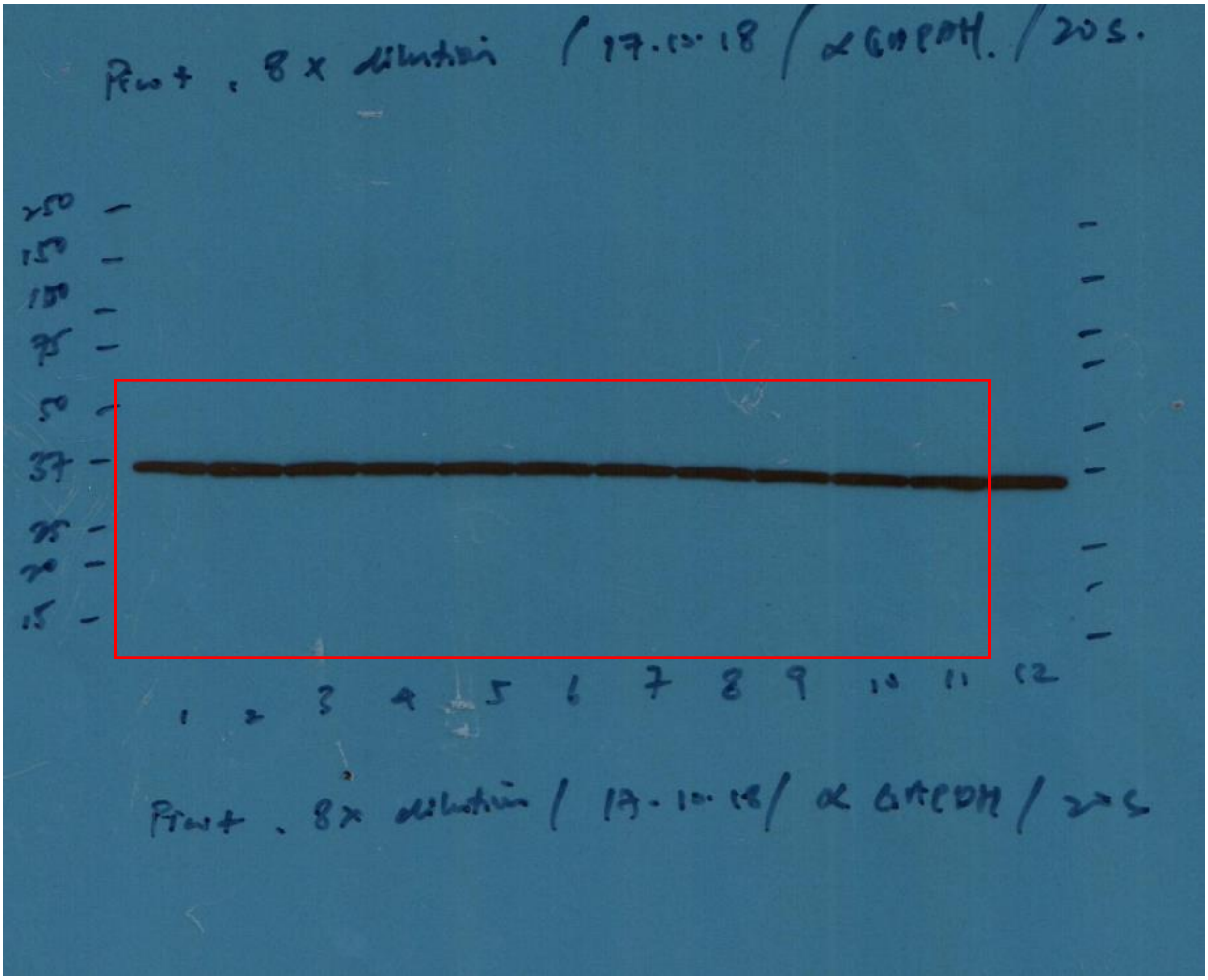

# Supplementary Figure 2f as seen in the manuscript

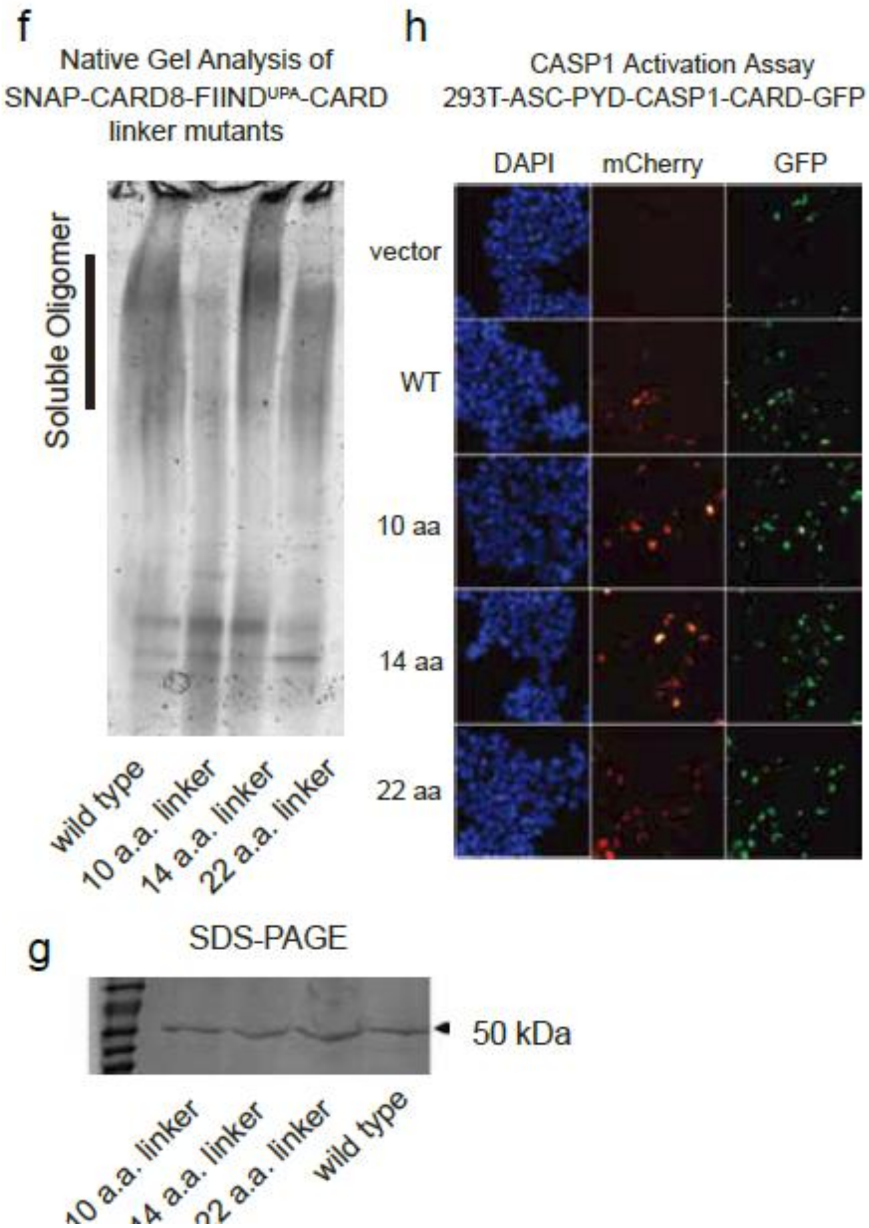

# Raw native Bis-Tris gel of Supplementary Figure 2f

CARD8 UPA linker mutants  UPA surface mutants

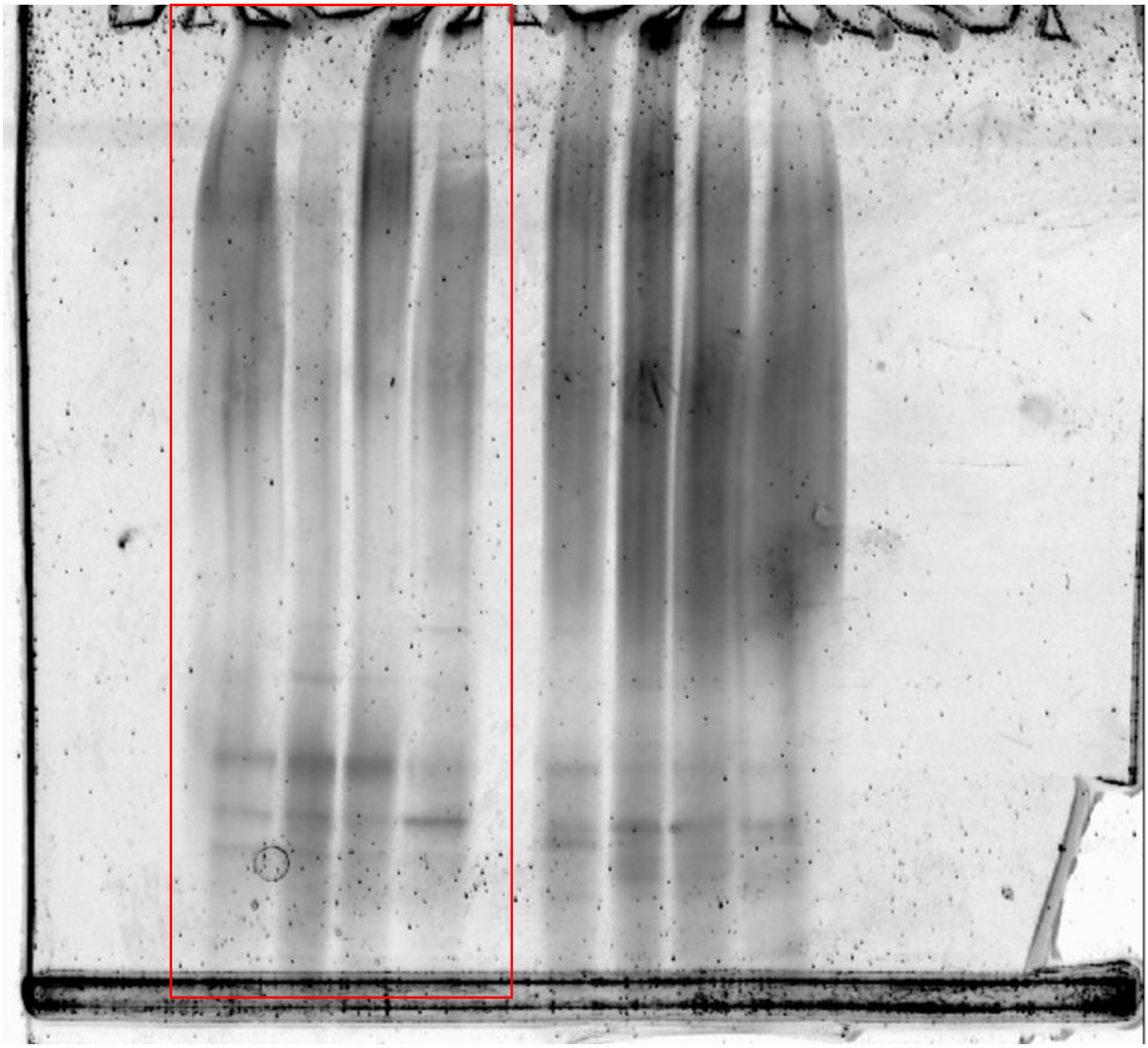

# Supplementary Figure 2g as seen in manuscript

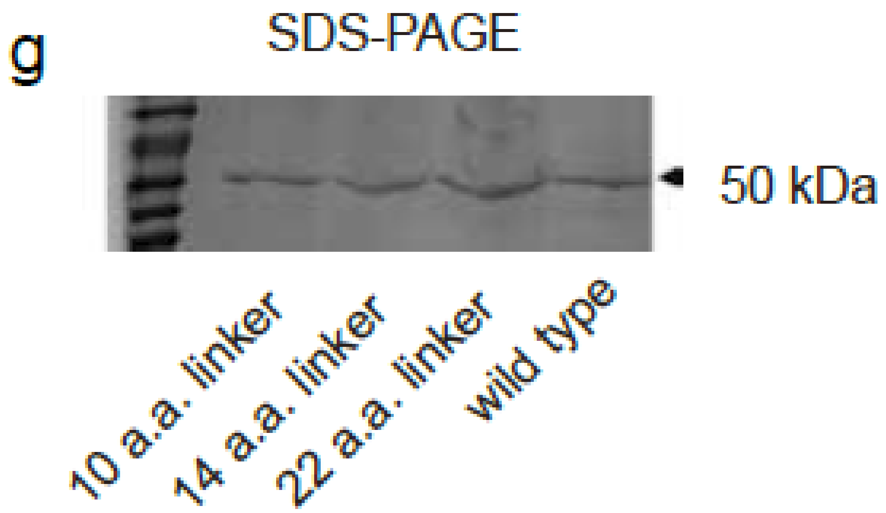

# Original SDS-PAGE image for Supplementary Figure 2g

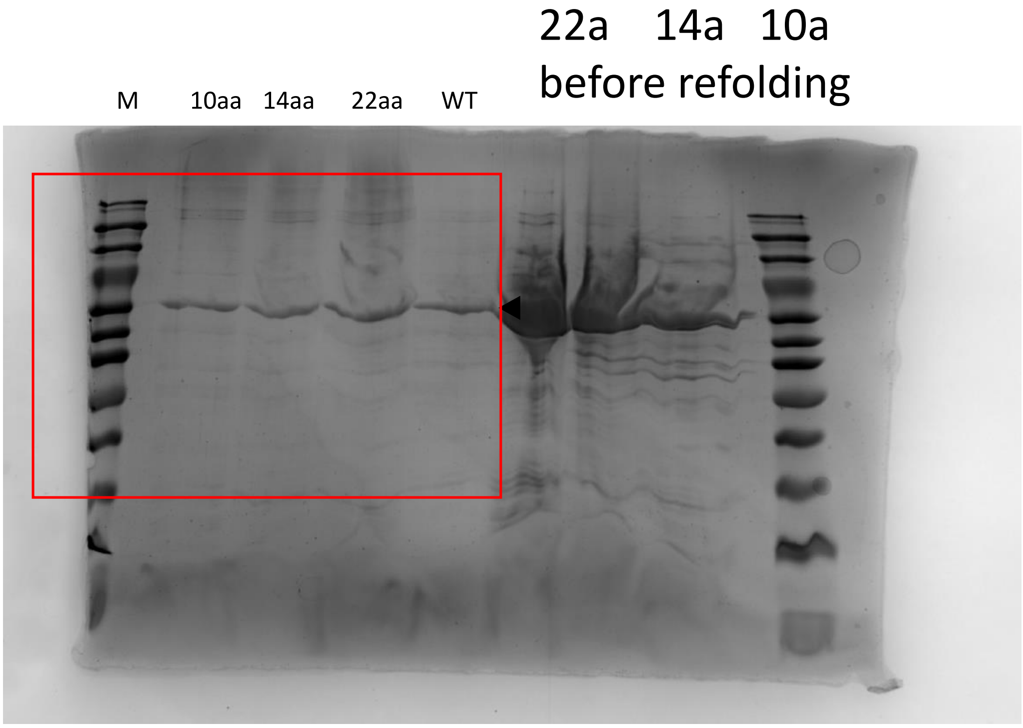

# Supplementary Figure 3a as seen in manuscript

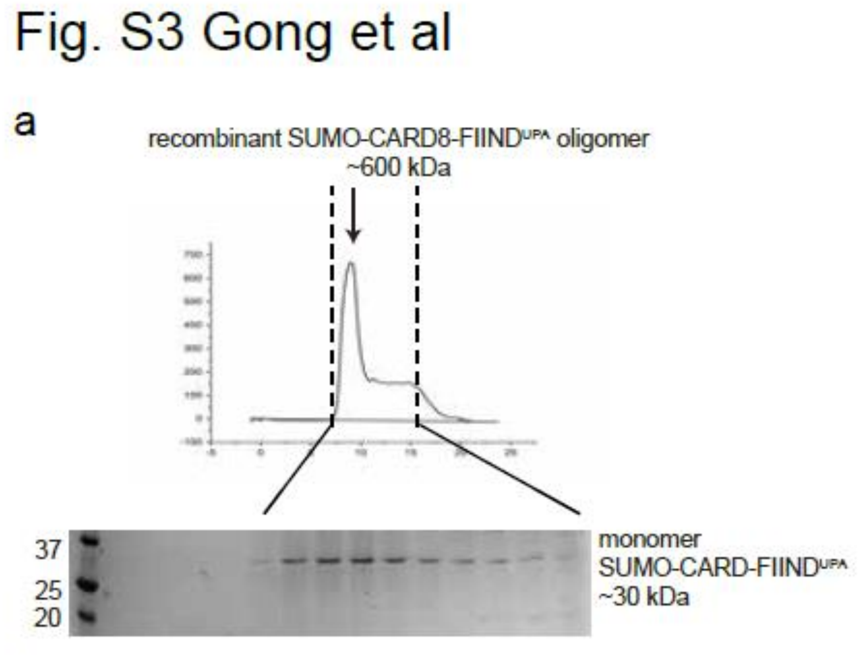

# Raw SDS-PAGE image of Supplementary Figure 3a

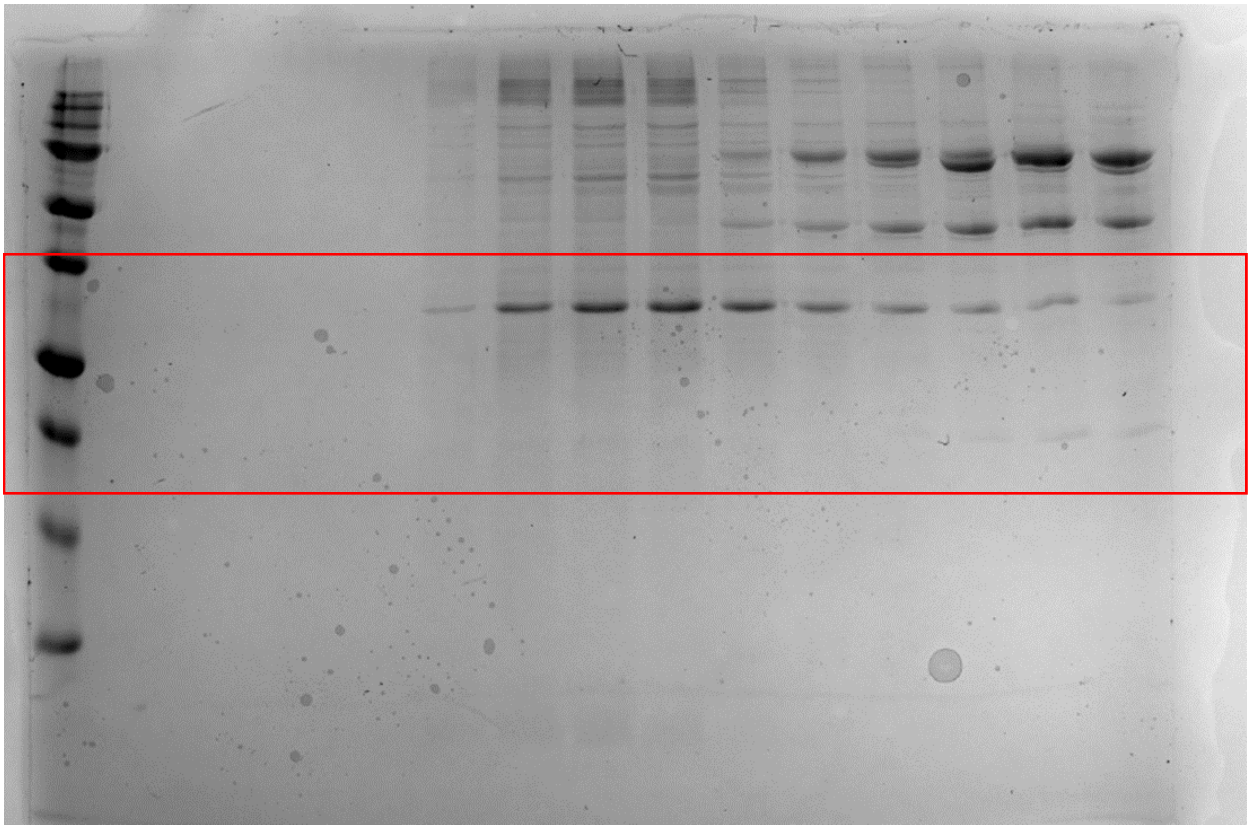

# Supplementary Figure 6f as seen in the manuscript

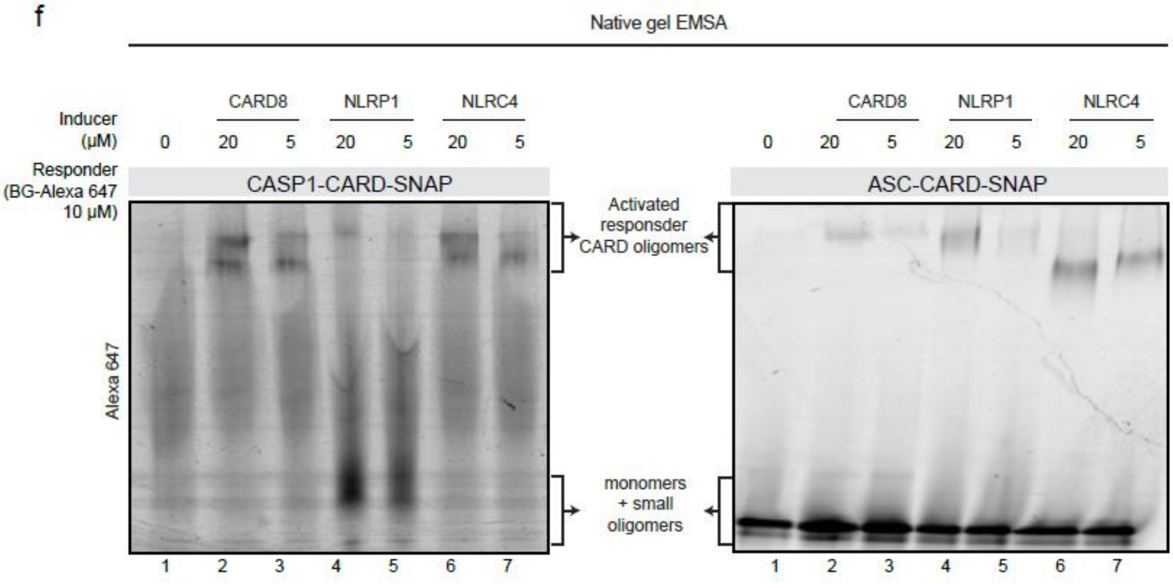

# Raw native BisTris-Tricine gel images for Supplementary Figure 6f

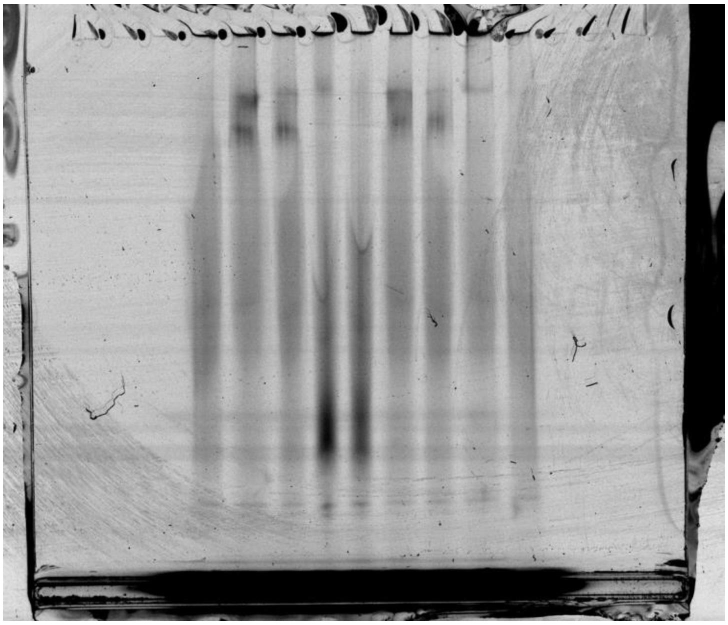

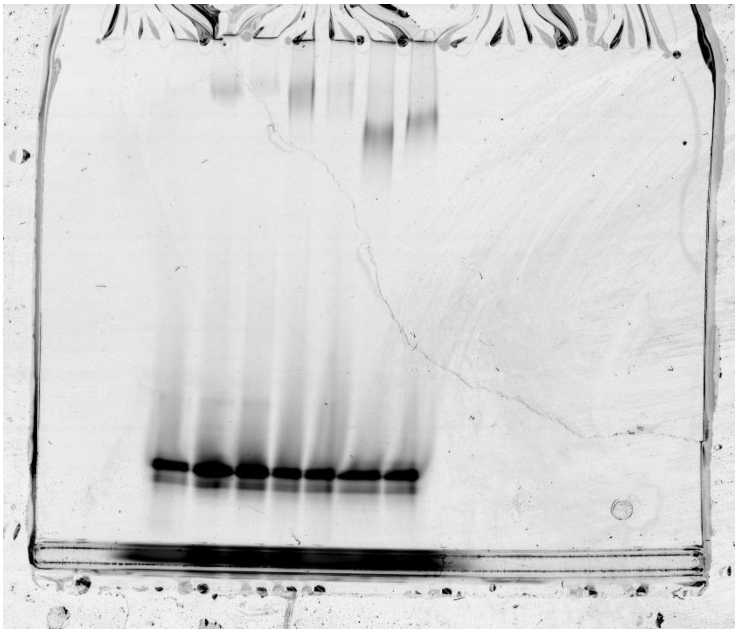

# Supplementary Figure 8d-e as seen in manuscript

Fig. S8 Gong et al

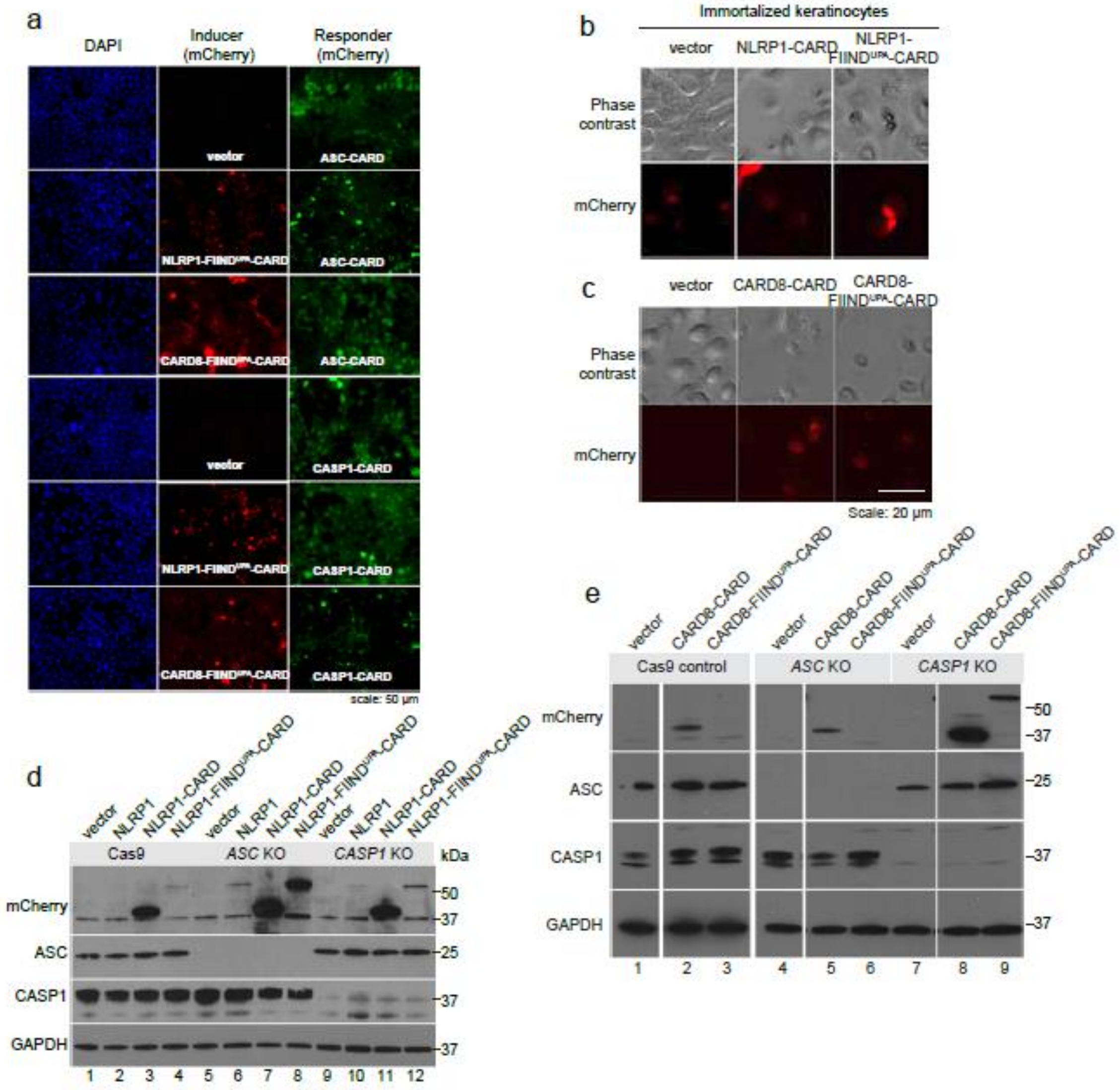

# Original Blot for Supplementary Figure 8d

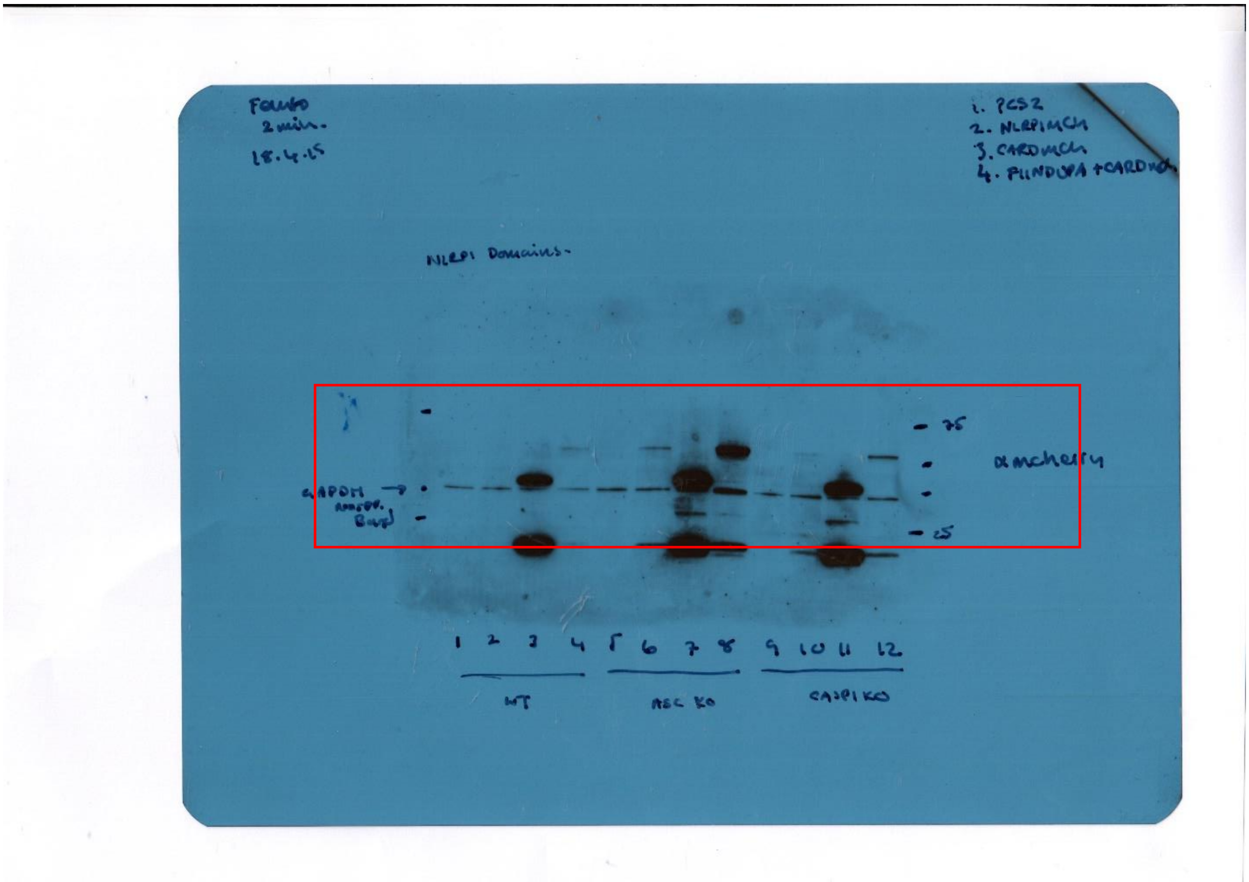

# Original Blot for Supplementary Figure 8d

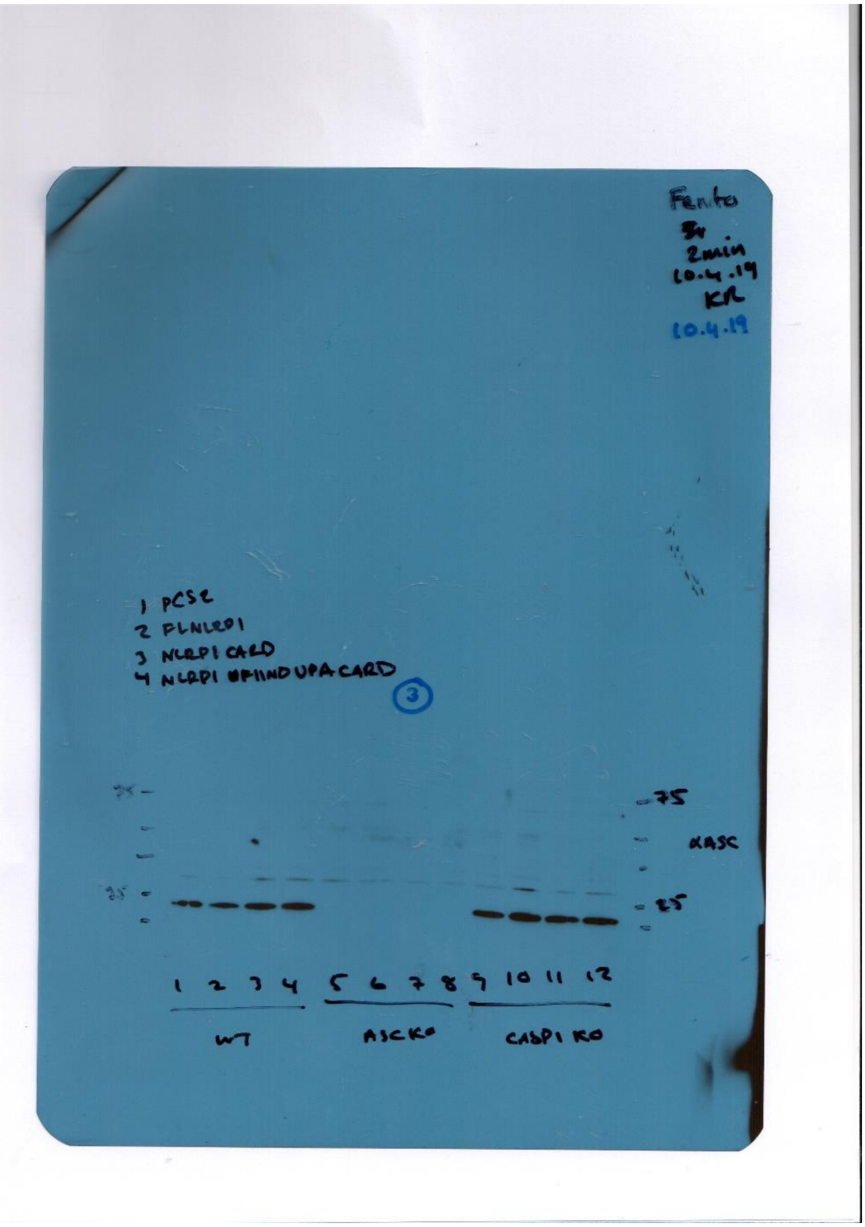

# Original Blot for Supplementary Figure 8d

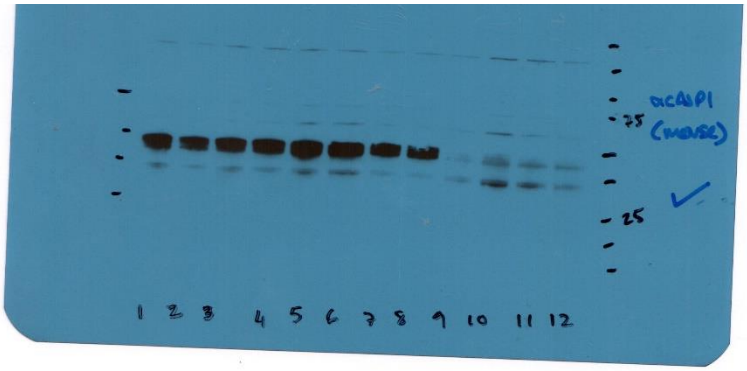


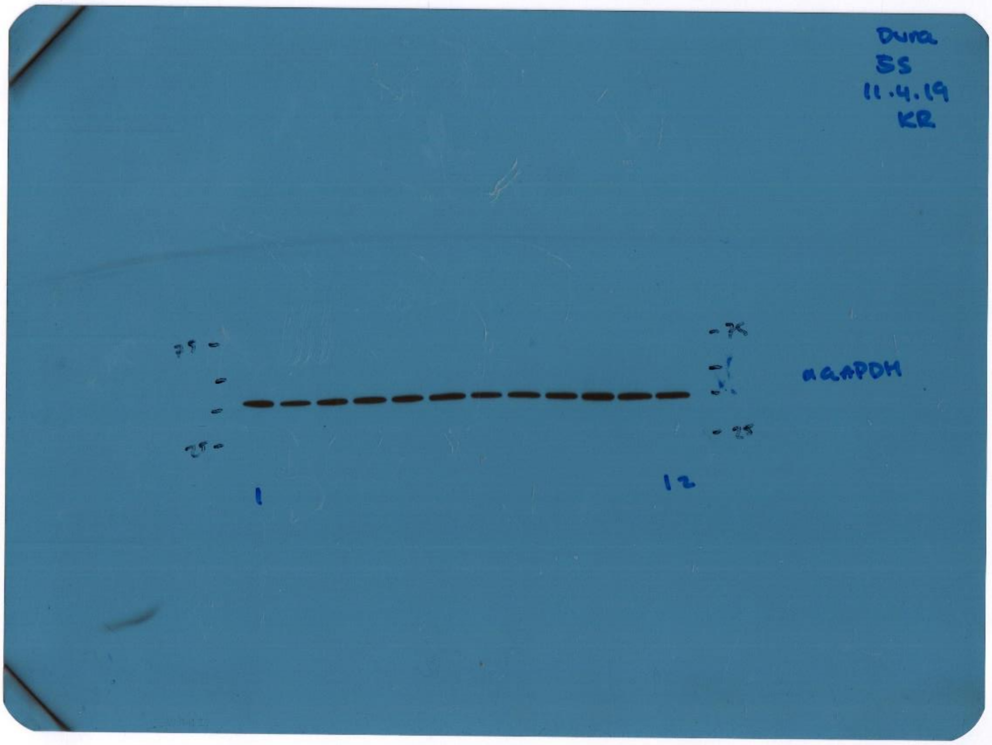

# Original Blot for Supplementary Figure 8e

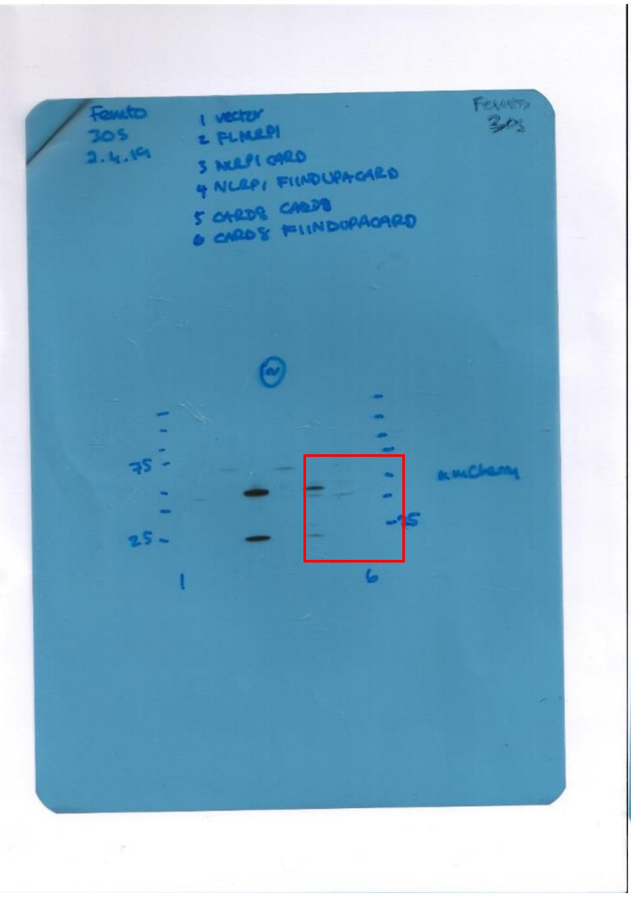


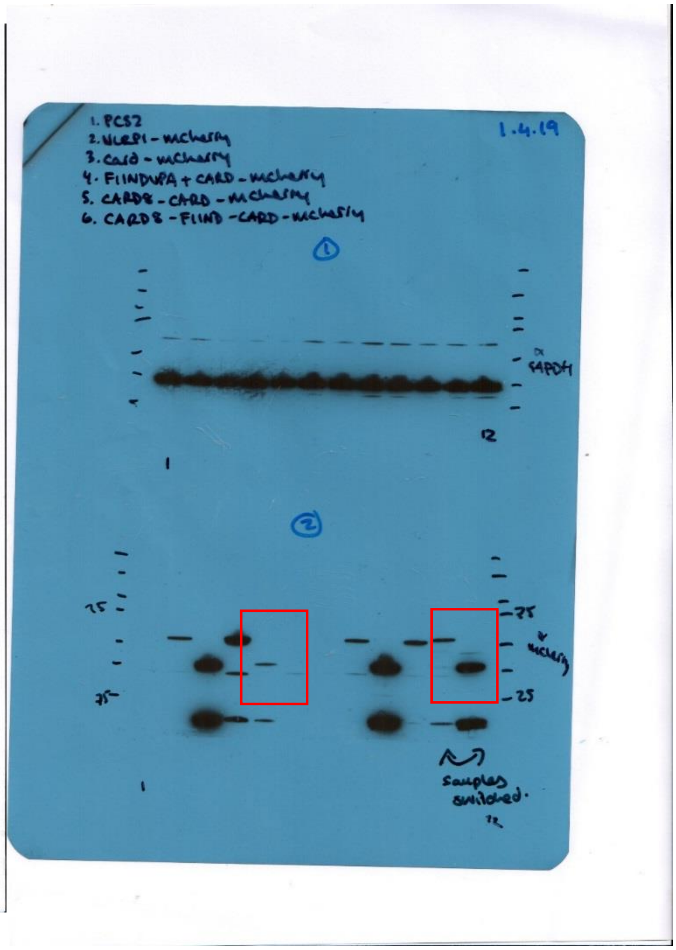

# Original Blot for Supplementary Figure 8e

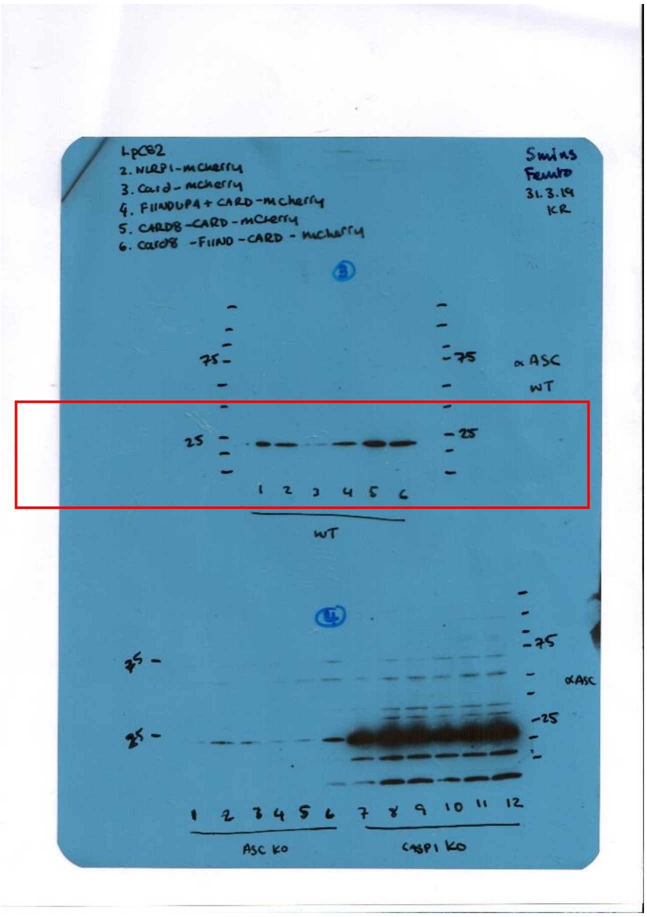


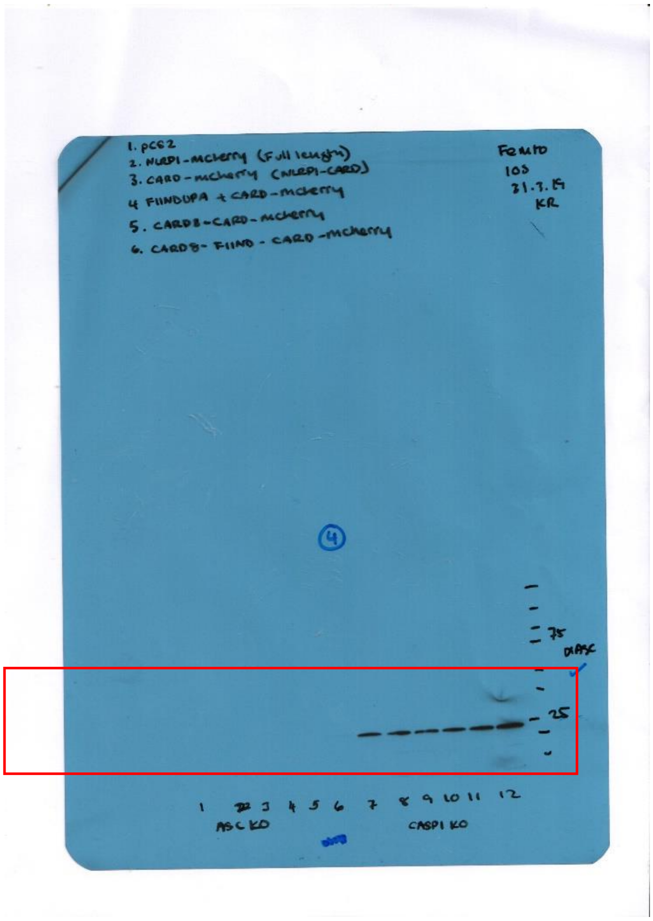

# Original Blot for Supplementary Figure 8e

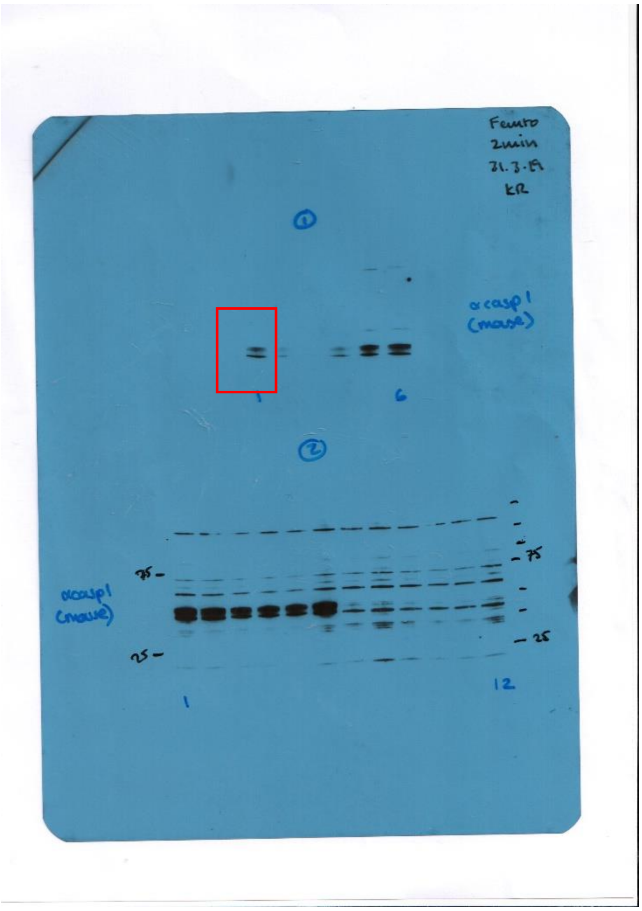

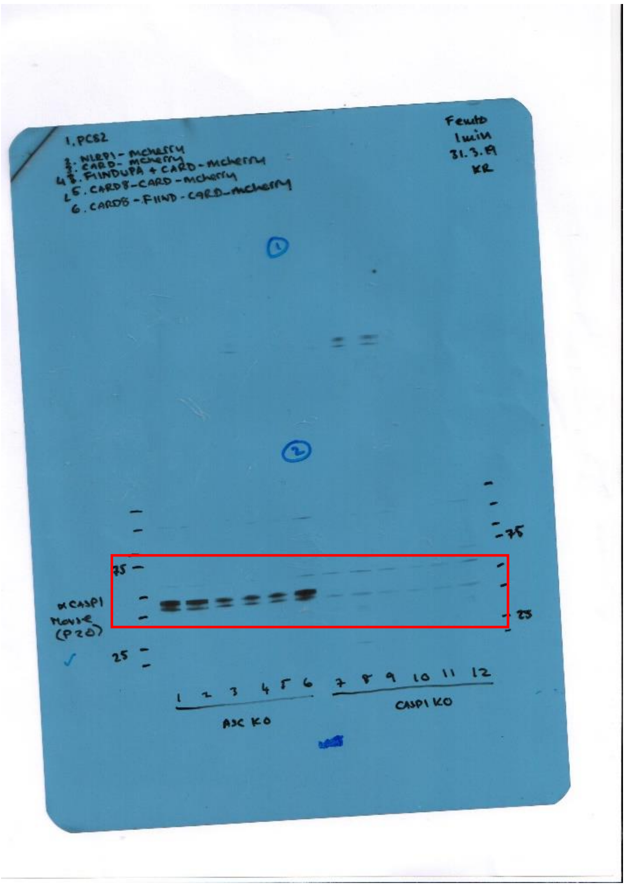

# Original Blot for Supplementary Figure 8e

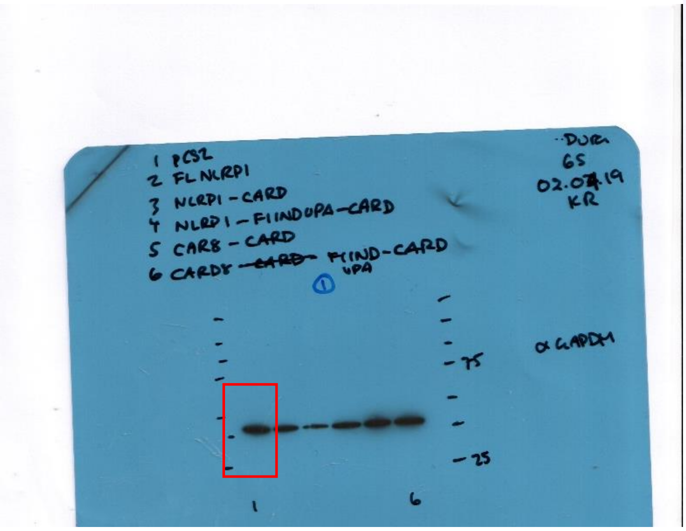

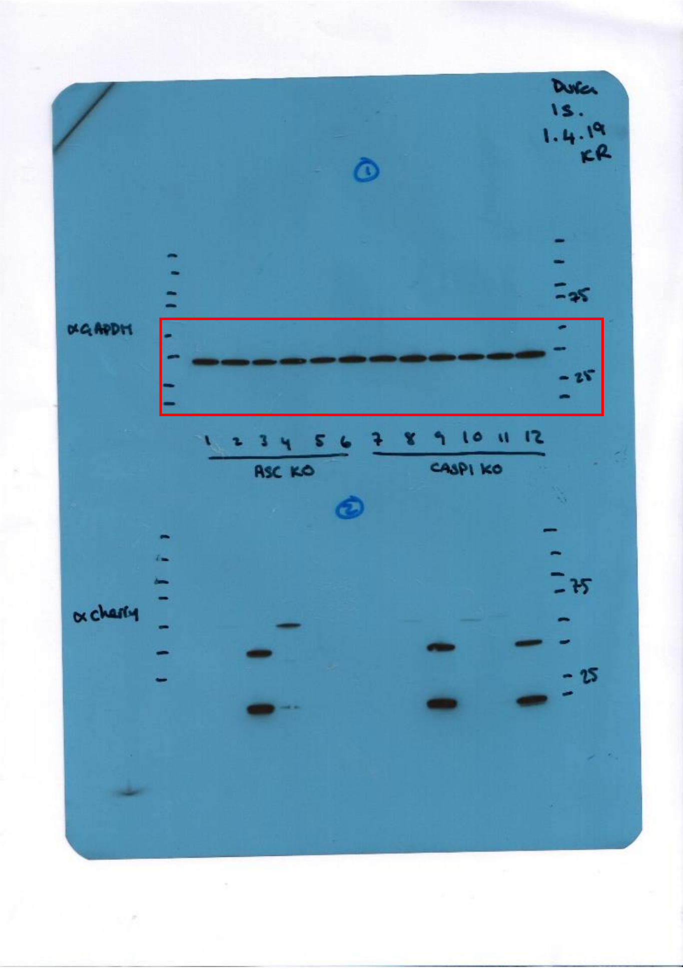

Supplement: Supplementary file 4 — Source Data [file 41467_2020_20319_MOESM4_ESM.zip › Additional_Source_Data_20201114_submission/Source data files_images_20201114.pdf]
